# Self-transformation of solid CaCO_3_ microspheres into core-shell and hollow hierarchical structures revealed by coherent X-ray diffraction imaging

**DOI:** 10.1107/S2052252522006108

**Published:** 2022-07-16

**Authors:** Thomas Beuvier, Yuriy Chushkin, Federico Zontone, Alain Gibaud, Oxana Cherkas, Julio Da Silva, Irina Snigireva

**Affiliations:** a LUNAM, IMMM, UMR 6283 CNRS, Faculté des Sciences, 72085 Le Mans Cedex 09 , France; b European Synchrotron Radiation Facility, 71 avenue des Martyrs, 38043 Grenoble Cedex 09, France; c Université Grenoble Alpes, CNRS, Grenoble INP, Institut Néel, 38000 Grenoble, France; University of Iowa, USA

**Keywords:** coherent X-ray diffraction, 3D X-ray fluorescence, microspheroids

## Abstract

The self-transformation of solid microspheres into complex core-shell and hollow architectures cannot be explained by classical Ostwald ripening alone. Here, coherent X-ray diffraction imaging and 3D X-ray fluorescence were used to visualize in 3D the formation of hollow microparticles of calcium carbonate in the presence of polystyrene sulfonate (PSS). During the dissolution of the core made from 10–25 nm crystals, the shell developed a global spheroidal shape composed of an innermost layer of 30 nm particles containing high PSS content on which oriented vaterite crystals grew with their *c* axis mainly oriented along the meridians. The stabilizing role of PSS and the minimization of the intercrystal dipolar energy can explain in combination with Ostwald ripening the formation of these sophisticated structures as encountered in many systems such as ZnO, TiO_2_, Fe_2_O_3_, Co_3_O_4_, MnO_2_, Cu_2_O, ZnS, CaCO_3_ and Ca_8_H_2_(PO_4_)_6_·5H_2_O.

## Abstract 2

Coherent X-ray diffraction imaging and 3D X-ray fluorescence were used to visualize in 3D the self-transformation of polycrystalline microspheres of calcium carbonate into hollow and core-shell microspheroid hierarchical structures.

## 1. Introduction

The common denominator between ice cream, nanometric emulsions, solar cells and age-hardenable aluminium alloy (Al–Cu) of fuselage skin in aircraft is that all these multiphase systems evolve during prolonged aging according to a mechanism called Ostwald ripening or coarsening (Ostwald, 1897 ▸). This thermodynamically driven phenomenon involves the transport of matter from smaller crystals to larger ones. The average size of solid or liquid particles thus tends to increase. In most applications, Ostwald ripening is considered a disadvantage. In ice cream, this yields a granular texture arising from the growth of ice crystals (Guo *et al.*, 2017 ▸). It is the main driving force in destabilizing oil/water emulsions of droplets below 150 nm (Delmas *et al.*, 2011 ▸). It also decreases the efficiency in some perovskite-based solar cells (Ummadisingu & Grätzel, 2018 ▸) and reduces the yield strength and hardness of age-hardenable aluminium alloys (Du *et al.*, 2017 ▸).

Yet Ostwald ripening under controlled manipulation can be fruitfully used to obtain hollow microspheres from solid ones as observed in ZnO (Fang *et al.*, 2012 ▸), TiO_2_ (Ye *et al.*, 2010 ▸), α-Fe_2_O_3,_(Xu & Zhu, 2011 ▸; Cao & Zhu, 2008 ▸), MnO_2_ (Jin *et al.*, 2010 ▸), Cu_2_O (Sui *et al.*, 2009 ▸), ZnS, Co_3_O_4_ (Liu & Zeng, 2005 ▸), Ca_8_H_2_(PO_4_)_6_·5H_2_O (Bigi *et al.*, 2002 ▸), and amorphous (Xu *et al.*, 2005 ▸) or crystallized CaCO_3_ (Yu *et al.*, 2003 ▸). The polycrystalline microspheres initially formed and filled with nanocrystals evolve into hollow microspheres by Ostwald ripening through an intermediate state labeled a homogeneous core-shell structure. Between the core and the shell, the gap formed is enlarged by transporting material from the less stable core to the more stable shell by Ostwald ripening. Once the core is completely dissolved, the hollow sphere is formed. The higher stability of nanocrystals in the shell compared with those in the core is generally attributed to either their larger size (Jin *et al.*, 2010 ▸; Sui *et al.*, 2009 ▸; Liu & Zeng, 2005 ▸) or their thermodynamically more stable phase (Xu *et al.*, 2005 ▸; Yu *et al.*, 2003 ▸, 2006 ▸). However, the mechanism of formation of such complex structures cannot be explained by Ostwald ripening alone. Various studies show (without any explanation) the presence of large holes on the outer surface of the microspheres (Ye *et al.*, 2010 ▸; Yu *et al.*, 2006 ▸; Cao & Zhu, 2008 ▸; Jin *et al.*, 2010 ▸; Sui *et al.*, 2009 ▸). In addition, the shell is usually composed of co-oriented nanocrystals (Bigi *et al.*, 2002 ▸) and sometimes has a spheroidal shape (Yu *et al.*, 2006 ▸; Gao *et al.*, 2006 ▸) instead of a spherical one. In some chemical systems, the shell exhibits a radial structure with large nanoparticles on the outer side and smaller nanoparticles on the inner side (Yu *et al.*, 2003 ▸), leading to a smooth appearance of the inner side (Bigi *et al.*, 2002 ▸).

How does such a hierarchical organization occur? Why does the growth of nanoparticles in the shell take place towards the outside and not the inside of the microstructures? Is there a link between the flattened morphology of the shell, the presence of holes at the surface of the shell and the co-alignment of the nanocrystals comprising the shell? Are there physical processes other than the Ostwald ripening that force the system to organize itself and what is the role of additives in such self-organization? To answer these questions, coherent X-ray diffraction imaging (CXDI) and X-ray fluorescence (XRF) tomography were used to 3D image single CaCO_3_ vaterite microspheres.

## 2. Results

### 2.1. Synthesis

All syntheses were carried out using a method similar to that reported by Yu *et al.* (2006 ▸). The precipitation of CaCO_3_ was carried out in glass vessels at room temperature (*ca* 22°C). Aqueous solutions of Na_2_CO_3_ (0.5 *M*) and CaCl_2_ (0.5 *M*) were first prepared as stock solutions. In a typical synthesis, a solution of CaCl_2_ (0.5 *M*, 0.165 ml for the 8 m*M* synthesis, 0.682 ml for the 30 m*M* synthesis, 4.615 ml for the 120 m*M* synthesis) was injected into an aqueous solution of polystyrene sulfonate (PSS; 10 ml, 0.24 to 8.0 g l^−1^). This formed CaCl_2_–PSS solutions. Then a solution of Na_2_CO_3_ (0.5 *M*, 0.165 ml for the 8 m*M* synthesis, 0.682 ml for the 30 m*M* synthesis, 4.615 ml for the 120 m*M* synthesis) was injected quickly into the CaCl_2_–PSS solution. After 1 min of stirring, this suspension was placed in an oven at 70°C for a specified reaction time for the recrystallization to take place (see Methods[Sec sec4]). Three parameters were studied: the equimolar calcium and carbonate concentration (8 m*M*, 30 m*M*, 120 m*M*), the PSS concentration (ranging from 0.24 to 8.0 g l^−1^) and the reaction time at 70°C without stirring (from 0 to 3 days). Therefore, we adopted the *XYZ* notation for each synthesis, where *X* corresponds to the CaCl_2_ concentration (equal to Na_2_CO_3_), *Y* corresponds to the PSS concentration in the CaCl_2_–PSS solution and *Z* refers to the reaction time at 70°C. For example, the synthesis 30 m*M* 1 g l^−1^ 30 min indicates the CaCl_2_ and Na_2_CO_3_ concentrations were 30 m*M*, the PSS concentration was 1 g l^−1^ in the CaCl_2_–PSS solution and the reaction time at 70°C was 30 min. Note that without any additive, vaterite would entirely transform into aragonite at 70°C in less than 2 h (Cherkas *et al.*, 2018 ▸).

### 2.2. Morphological features of the hollow microspheroids

**Figure 1:**
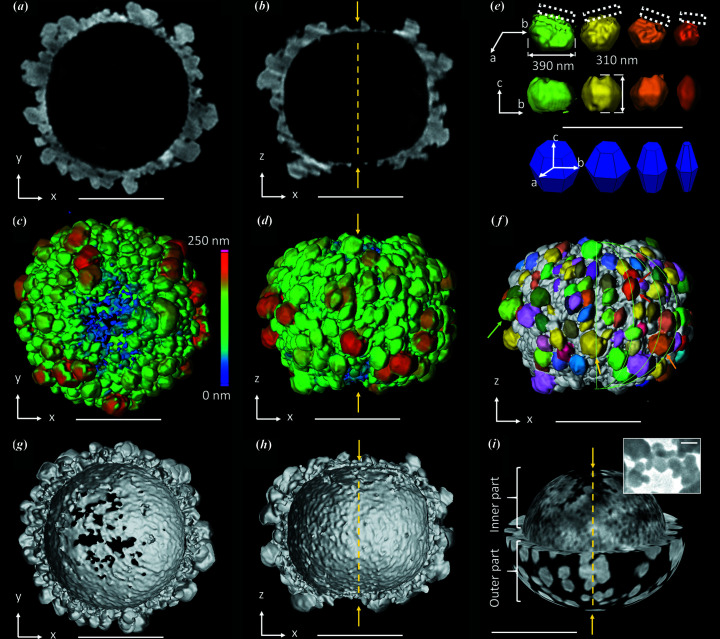
Morphological features of the hollow microspheroids in the final stages. CXDI of vaterite microspheroids obtained from the 8 m*M* 1 g l^−1^ 24 h synthesis. (*a*) and (*b*) CXDI 2D views through the center of the microspheroid. The yellow arrows indicate the poles. (*c*) and (*d*) 3D views showing the wall thickness of the shell, thinner at the poles, thicker at the equator. (*e*) Selected vaterite nanocrystals extracted from the shell of the microspheroid. Note that the crystallographic structure of vaterite is still controversial (Christy, 2017 ▸). Here, the vectors *a*, *b* and *c* refer here to the crystallographic axes of vaterite according to Kamhi’s model [*P*6_3_/*mmc* space group (Kamhi, 1963 ▸)]. The dotted rectangular line represents the inner part of the shell. (*f*) 3D view of the microspheroid where around 100 vaterite crystals were segmented. Those shown in (*e*) are marked with an arrow. They all display a truncated hexagonal based bipyramidal shape, many of them having their *c* axis oriented along the meridians. (*g*) and (*h*) 3D views showing half of the microspheroid. (*i*) Spheroidal 2D views of the shell showing the juxtaposition of two layers made of (bottom) large crystals on the outer part and (top) small crystals close to 30 nm on the inner part of the shell. In the inset, a TEM image of nanoparticles of the poles belonging to the inner part of the shell is shown. Scale bar = 1 µm except in the inset of (*g*) for which the scale bar is 50 nm. Note here that S:Ca = 0.59.

Fig. 1 ▸ shows the typical morphology observed by CXDI of a microparticle formed from 8 m*M* 1 g l^−1^ solutions after 24 h. This illustrates the final hierarchical structure of the microparticle characterized by a completely hollow core [Figs. 1 ▸(*a*) and 1 ▸(*b*)], a shell with a oblate spheroidal morphology, and with thicknesses between 0 in the region of the poles and around 250 nm at the equator [Figs. 1 ▸(*c*) and 1 ▸(*d*)]. The outer surface of the shell is decorated by crystals of a few hundred nanometres in size in the form of hexagonal based bipyramids [Fig. 1 ▸(*e*)] mainly oriented along the meridians [Fig. 1 ▸(*f*)]. Spheroidal 2D views of the outer part of the shell [bottom of Fig. 1 ▸(*i*)] and of its inner part [top of Fig. 1 ▸(*i*)] highlight the morphological difference between the inner and the outer parts of the shell. A fine analysis shows that the inner part of the shell is composed of nanodomains of ∼30 nm, as confirmed by TEM images obtained close to the pole [inset of Fig. 1 ▸(*i*), see details in Fig. S1].

### 2.3. Formation of an ultra-thin shell

**Figure 2:**
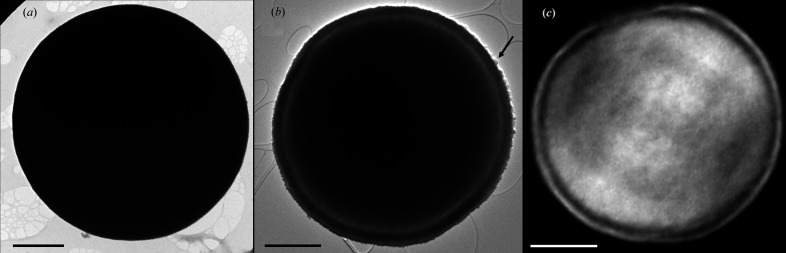
Microspheres in the early stages. (*a*) and (*b*) TEM images of vaterite microspheres obtained from the syntheses (*a*) 8 m*M* 1 g l^−1^ 1 min (*i.e.* after 1 min of reaction) and (*b*) 8 m*M* 1 g l^−1^ 30 min (*i.e.* after 30 min of reaction). The black arrow shows a crystal of around 60 nm width. (*c*) CXDI 2D view of 8 m*M* 1 g l^−1^ 30 min. Scale bar = 1 µm. Note S:Ca = 0.59.

It is intriguing to understand how such a complex morphology can be formed during direct precipitation of the initial precursors. To unravel the mechanism, we monitored the morphological evolution of these microparticles synthesized at lower reaction times (*i.e.* 1 min, 30 min). After 1 min, it is clear from the TEM images shown in Fig. 2 ▸(*a*) that microspheres appear completely full as expected from the direct precipitation of precursors. After 30 min of reaction, the presence of a shell separated from the core becomes quite visible with nanocrystals having a maximum size of around 60 nm at the outer surface of the shell [see the black arrow in Fig. 2 ▸(*b*)]. Even though we could not assess the morphology of these crystals with CXDI due to the too-low ratio of crystal size to voxel size (voxel size = 32 nm here), the presence of an ∼100 nm-thick shell is confirmed. Even if the packing of the nanoparticles is too high to determine the sizes of the nanoparticles, a thickness of 100 nm may correspond to a stack of at least three nanoparticles 30 nm thick. By comparing the TEM images after 1 and 30 min of reaction, we can conclude that the crystals of vaterite at the outer surface of the microspheres grew in the first 30 min of reaction, probably from Ca^2+^ and CO_3_^2−^ ions of the solution, the outermost layers being in direct contact with the solution. The nanocrystals may also grow in a second step from the Ca^2+^ and CO_3_^2−^ ions from the dissolution of smaller crystals just below this outer layer. This latter process marks the beginning of the Ostwald ripening with the dissolution of the small particles at the benefit of the larger ones. Note that the *in situ* infrared spectroscopy shown in Fig. S2 demonstrates that vaterite appears within 1 min of reaction. Furthermore, no amorphous calcium carbonate (ACC) was detected. This suggests that the main mechanism explaining the formation of a core-shell structure is the Ostwald ripening and probably not a phase transformation from ACC to vaterite.

### 2.4. Formation of a thick flattened shell with holes at the poles

**Figure 3:**
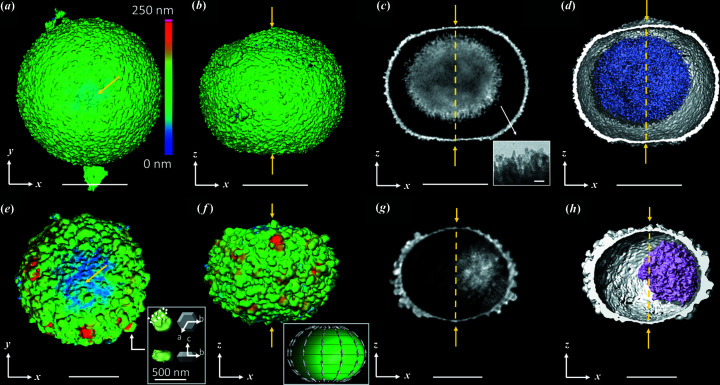
Oblate microspheroids in the intermediate stages. CXDI images of vaterite microspheroids obtained from the syntheses (*a*)–(*d*) 8 m*M* 1 g l^−1^ 2 h and (*e*)–(*h*) 8 m*M* 1 g l^−1^ 8 h with (*a*), (*b*), (*e*) and (*f*) 3D views showing the wall thickness of the shell from 0 to 250 nm. (*c*) and (*g*) CXDI 2D slices viewed in the *xz* plane. The inset of (*c*) shows a TEM image of the edge of a core. This allows access to the size of the nanoparticles of the core (details provided in Fig. S3). (*d*) and (*h*) 3D views of the core depicted in blue, with half the shell cut away to allow observation of the inside. The yellow arrows indicate the poles. In (*e*), the inset shows vaterite crystals extracted from the microspheroid (in green) and the platelet-associated crystal geometry (in white). The vectors *a*, *b* and *c* refer here to the crystallographic axes of vaterite according to Kamhi’s model [*P*6_3_/*mmc* space group (Kamhi, 1963 ▸)]. The inset of (*f*) displays a scheme of the microspheroid. The arrows represent the dipole moments associated with the vaterite nanoparticles. The poles are free of nanoparticles to reduce the dipole energy. Note S:Ca = 0.59. Scale bar = 2 µm for (*a*)–(*d*) and 1 µm for (*e*)–(*h*).

After 2 and 8 h of reaction, the microparticles display several interesting features (Fig. 3 ▸). The microspheres flatten out at the poles (tracing the *z* direction and marked by yellow arrows in Fig. 3 ▸) and retain a circular symmetry equatorially (in the orthogonal *xy* plane). The poles on the shell are slightly visible after 2 h of reaction but can be clearly identified after 8 h. The microparticles are thus like oblate spheroids. The thickness of the shell over the entire surface was determined. It is clearly minimal at the poles [Figs. 3 ▸(*a*) and 3 ▸(*e*)] in line with our previous observations [Fig. 1 ▸(*c*) and 1 ▸(*d*)]. After 2 h of reaction, TEM reveals also that the outer part of the shell contains 80–100 nm nanoparticles (Fig. S3) which grow up to 300 nm after 8 h of reaction as shown by CXDI [inset of Figs. 3 ▸(*e*) and S4]. Their segmentation from CXDI images highlights a hexagonal shape typical of the vaterite phase, and similar to the hexagonal platelets commonly found in the literature (Wang *et al.*, 2015 ▸; Zhu *et al.*, 2009 ▸; Gehrke *et al.*, 2005 ▸). This strongly points to a crystallographic *c* axis directed about the normal of the hexagonal faces, and is confirmed by TEM. Note that these normal vectors are mainly oriented along the meridians as shown schematically in the inset of Fig. 3 ▸(*f*) and detailed in Fig. S4, even though the alignment shows some dispersion. As observed in Fig. 1 ▸ for particles obtained after 24 h of reaction, this reflects a link between (1) the co-alignment of crystals of the shell, (2) the oblate spheroidal shape of the shell and (3) the thinning of the shell at the poles. Therefore the system evolves over time towards an organized arrangement of nanoparticles with, at the poles, a thinning of the shell. Many studies propose that the organized assembly of vaterite nanoparticles into mesostructures has its origin in the dipole interactions between nanoparticles (Xu *et al.*, 2014 ▸; Wang *et al.*, 2015 ▸; Zhu *et al.*, 2009 ▸). Two dipole moments have the lowest dipole energy when they are parallel. It is therefore possible that an organization of nanoparticles within the microspheres exists after 30 min of ripening but that it is not observed by CXDI at the current resolution. The poles can thus be considered as frustrated regions where the complete alignment of the dipoles is not possible. The nanoparticles of the poles tend to dissolve due to their high dipole energy yielding with time to depleted poles with large holes. In Figs. 3 ▸(*c*) and 3 ▸(*g*), we can see that the core/shell morphology is now fully developed similar to MnO_2_ (Jin *et al.*, 2010 ▸), Cu_2_O (Sui *et al.*, 2009 ▸), ZnS and Co_3_O_4_ (Liu & Zeng, 2005 ▸) microspheres commonly reported in the literature.

### 2.5. Dissolution of the core

After 2 h, the core resembles a dendritic ‘hairy’ sphere composed on its outer part of nanoparticles ranging from 10 to 30 nm [inset of Fig. 3 ▸(*c*), see details in Fig. S3]. Their sizes are thus equal to or smaller than those of the nanoparticles that comprise the inner part of the shell [Fig. 1 ▸(*i*)] and much less packed. We propose that the formation of a gap between the shell and the core takes place because the nanoparticles in the inner part of the shell are slightly more stable than those of the core, due to either their higher sizes or the higher content of PSS, or both. After 8 h of reaction, the core becomes drastically smaller due to progressive dissolution with time concomitantly with the growth of vaterite crystals on the outer surface of the shell. Two CXDI images obtained of two other microspheroids after 8 h of reaction are shown in Fig. S5. They display similar sizes and features with a oblate spheroidal shape of the shell with hexagonal based vaterite crystals oriented outwards and a ‘hairy’ core made of radially distributed nanoparticles. This highlights that the selected particles observed by CXDI are representative of the whole population (also confirmed by SEM and TEM observations, see Fig. S6). As previously mentioned, the inner surface of the shell still appears smooth at our resolution (voxel size = 32 nm for 8 m*M* 1 g l^−1^ 2 h, voxel size = 17 nm for 8 m*M* 1 g l^−1^ 8 h) [Figs. 3 ▸(*d*) and 3 ▸(*h*)] in stark contrast to the rough ‘hairy’ surface of the core (represented in blue on these same figures). The smooth appearance originates from the tightly packed nanoparticles of ∼30 nm in the inner part of the shell, while the hairy appearance of the core reflects that some nanoparticles dissolve. Finally, we observed an unexplained slight reduction in the diameter of the microspheroids with time.

### 2.6. Mechanism of shell formation

**Figure 4:**
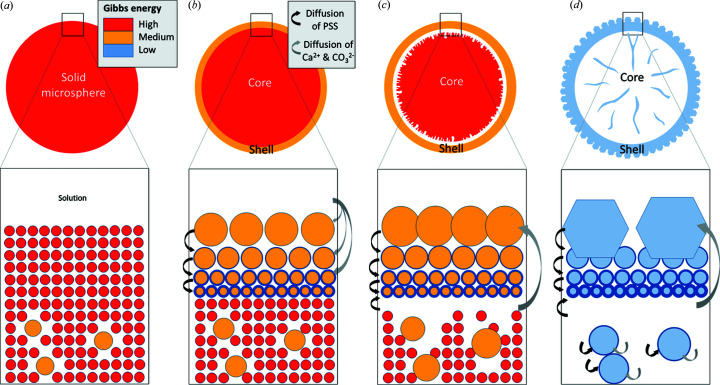
Self-transformation of a solid microsphere (made of highly monodisperse size nanoparticles) into a core-shell structure. (*a*) Formation of a solid microsphere made from highly monodisperse size nanoparticles of high Gibbs free energy. The color of the nanoparticles refers to their Gibbs free energy (*G*) with, for simplicity, three levels: red (high *G*), orange (medium *G*) and blue (low *G*). (*b*) Formation of a multilayered shell shown in orange. The outermost layers are more stable due to their larger nanoparticles. The innermost layers are more stable due to their lower interfacial free energy. The higher thickness of the contour of the nanoparticles refers to a higher PSS coverage corresponding to a lower interfacial free energy. In the core, the largest nanoparticles continue to grow by Ostwald ripening. (*c*) Progressive dissolution of the core forming a core-shell structure. (*d*) Formation of an almost-hollow microsphere. The presence of nanoparticles in the core results from the growth of the largest nanoparticles of the initial solid microspheres. With full size monodispersity of the nanoparticles in the initial solid microsphere, the final structure would be completely hollow.

How can we explain the architecture of the shell with nanoparticles of very different sizes [∼30 nm in the inner part of the shell even after 72 h of reaction (Fig. S1) and >300 nm in the outer part] and why nanoparticles of the inner shell do not grow inward (toward the center)? These two questions are linked and can be summarized by the following question: why are nanoparticles in the inner layer of the shell stable and do not tend to grow or dissolve? We propose that in the first moments of formation of the solid microspheres, PSS macromolecules were trapped, probably adsorbed on the surface of all the vaterite nanoparticles in the entire volume of the microspheres [Fig. 4 ▸(*a*)]. Then, the growth of the outermost layer of the microspheres by the addition of ions or clusters from the supersaturated solution expels PSS [Fig. 4 ▸(*b*)]. Some macromolecules remained in the solution, while others were adsorbed to the underneath of the nanoparticle. This process created, in the inner part of the shell, a layer of nanoparticles of ∼30 nm, rich in PSS, delimiting the core from the shell. Another possible origin of the PSS in the inner shell is the dissolution of the core. During this process, PSS is released and may diffuse to the inner shell and coat the nanoparticles [Figs. 4 ▸(*c*) and 4 ▸(*d*)].

### 2.7. Observation of PSS inside the microsphere

**Figure 5:**
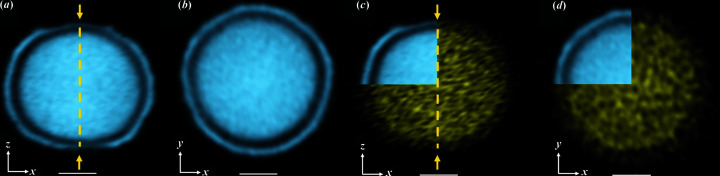
Distribution of calcium and sulfur in the microspheres. 3D-XRF images of 8 m*M* 1 g l^−1^ 30 min with (*a*) a calcium *xz* plane, (*b*) a calcium *xy* plane, (*c*) a sulfur *xz* plane and (*d*) a sulfur 2D *xy* plane. On (*c*) and (*d*), the upper left corner of the image corresponds to calcium. This allows the sulfur and calcium signals to be visualized on the same image. Scale bar = 1 µm. Note S:Ca = 0.59.

To probe whether the microspheres contain PSS, 3D XRF tomography with a voxel size of 65 nm was carried out on a core-shell microspheroid of 8 m*M* 1 g l^−1^ 30 min. Figs. 5 ▸(*a*) and 5 ▸(*b*) show the distribution of calcium on the *xz* plane and equatorial (*i.e. xy* plane) cross-sections; Figs. 5 ▸(*c*) and 5 ▸(*d*) show the corresponding distribution of sulfur belonging to the PSS. There are two interesting points to note. First, the calcium fluorescence signal confirms the core-shell architecture and the oblate spheroidal shape of the shell with a reduced thickness at the poles [Figs. 5 ▸(*a*) and 5 ▸(*b*)]. Secondly, the presence of sulfur inside the microsphere proves that it contains PSS. This supports the mechanism proposed above considering that the nanoparticles of the inner part of the shell can be stabilized by PSS from either the growth of the outermost layer or the dissolution of the core, or both. Unfortunately, the resolution of 3D-XRF (voxel size of 65 nm) is not fine enough to access the repartition of PSS within the shell.

### 2.8. Effect of PSS concentration

**Figure 6:**
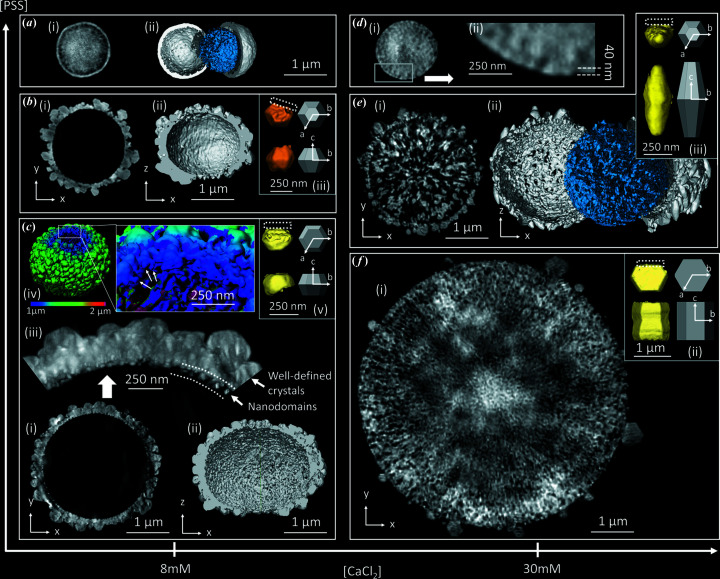
Effect of PSS and CaCl_2_ concentrations. CXDI images obtained on microparticles after long reaction times (≥24 h) for different initial concentrations of PSS (vertical axis) and CaCl_2_ (horizontal axis). (*a*) 8 m*M* 1.94 g l^−1^ 24 h (*V_x_* = 13.8 nm) with (i) a 2D view and (ii) a 3D view. The shell (white) was cut into two parts to reveal its smooth inner part. The core is blue. (*b*) 8 m*M* 1.0 g l^−1^ 24 h (*V_x_* = 16.4 nm) with (i) a 2D view and (ii) a 3D view of half the microspheroid. In inset (iii), a vaterite crystal (orange) extracted from the outer part of the shell is shown and compared with a truncated hexagonal base bipyramid. Detailed results are shown in Figs. 1 ▸(*e*) and 1 ▸(*f*). The same kind of segmentation can be seen in the insets of (*c*), (*e*) and (*f*). (*c*) 8 m*M* 0.27 g l^−1^ 24 h (*V_x_* = 12.6 nm) with (i) a 2D view, (ii) a 3D view of half of the microspheroid, (iii) a 2D view of a selected region and (iv) a 3D view revealing the nanostructure of the pole. The color corresponds to the distance of the surface to the center of the microspheroid. This allows us to highlight the spheroidal shape of the microparticles. A zoomed-in view at the pole allows us to identify the presence of individual nanoparticles of ∼30 nm. In (iii), the image is obtained by superimposing a stack of images oriented according to *z* and over a thickness of 400 nm. In each pixel of the resulting image, the grayscale is equal to the maximum grayscale of the pixels in the image stack (details in Fig. S12). (*d*) 30 m*M* 8.0 g l^−1^ 72 h (*V_x_* = 17.7 nm) with (i) a 2D view and (ii) a zoomed-in view of the white rectangular region. The thickness of the shell is difficult to observe and close to 40 nm. (*e*) 30 m*M* 1.0 g l^−1^ 72 h (*V_x_* = 17.7 nm) with (i) a 2D view and (ii) a 3D view. The shell (white) was cut into two parts to reveal the inner part of the shell. The core is blue. (*f*) 30 m*M* 0.27 g l^−1^ 24 h (*V_x_* = 32.7 nm) with (i) a 2D view and (ii) 3D views of the largest crystal of vaterite extracted from the outer part of the shell. In (i) the shell cannot be seen. *V_x_* = voxel size. The dotted rectangular line represents the region of the inner part of the shell.

Let us first compare the effect of an increasing concentration of PSS at a fixed concentration of CaCl_2_ (8 m*M*) after 24 h of aging. When the PSS concentration is increased from 1.0 g l^−1^ (case 1 corresponding to a molar ratio of S:Ca = 0.59) to 1.94 g l^−1^ (case 2 corresponding to a molar ratio of S:Ca = 1.14), the core remains almost complete even after 24 h of reaction time and the outer crystals of the shell are not developed [see Fig. 6 ▸(*a*)]. This result is in marked contrast with that observed after 24 h in case 1 where the core completely vanished [see Fig. 6 ▸(*b*)] with large crystals within the shell. We conclude that the dynamics of the Ostwald ripening are therefore greatly slowed down at higher PSS concentration. In other terms, once the microspheres are formed, the kinetics of the dissolution of the less stable nanoparticles and the growth of the most stable ones are reduced, probably because all the nanoparticles are covered with more PSS. However, PSS has a second effect. It also reduces the size of the microspheres, the smallest sizes of the microparticles being observed for the highest concentrations of PSS. This may result from the slower growth of each nanoparticle during formation of the microspheres at the early stage. By following the pH in the first minute of reaction with and without PSS, and by looking at the turbidity of the suspensions (Fig. S7), the total amount of solid CaCO_3_ formed is delayed in the presence of PSS. A similar effect was observed with polyaspartate and polyacrylate (carboxyl­ate groups) in the precipitation of calcium phosphate (Bigi *et al.*, 2002 ▸), or more prominently with phylic acid [phosphate groups (Xu *et al.*, 2005 ▸)].

Interestingly, we can ask what would happen if we decrease the S:Ca ratio even further. The experiments were thus conducted at 8 m*M* 0.27 g l^−1^ 24 h corresponding to an S:Ca molar ratio of 0.16, around 4× lower. This lower ratio is consistent with a much weaker influence of the PSS molecules. Again, we observe that the core has disappeared after 24 h [Fig. 6 ▸(*c*), parts (i) and (ii)]. Yet the inner surface of the shell is less smooth than for S:Ca = 0.59 probably because the number of PSS molecules carpeting the inner surface becomes insufficient to protect the nanoparticles from dissolution. CXDI allows us to estimate the size of the nanoparticles of the inner surface at about 30 nm [Fig. 6 ▸(*c*) parts (iii) and (iv)]. This value is confirmed by SEM observations (Fig. S8) and is similar to that observed by TEM for 8 m*M* 1 g l^−1^ 24 h [inset Fig. 1 ▸(*i*)]. These observations confirm the coexistence of nanoparticles of different sizes within this shell: on the outer part, nanoparticles of a few hundred nanometres; on the inner part, nanoparticles of about 30 nm. The juxtaposition of crystals of different sizes spread on two well defined layers cannot be explained by considering only the Ostwald ripening mechanism, according to which the smallest particles should rapidly dissolve. The kinetic stabilization is likely to result from the presence of PSS as explained in Fig. 4 ▸.

We can conclude from these experiments that the S:Ca ratio controls the ripening kinetics of the microspheres. As we will see now, the scenario is not so simple. The morphology of the particles depends also on the initial supersaturation. We now demonstrate this using almost the same S:Ca ratio than in the aforementioned experiment but by increasing the calcium and carbonate concentrations of the precursors to 30 m*M* instead of working at 8 m*M*.

### 2.9. Effect of initial Ca^2+^ and CO_3_^2−^ concentrations

Figs. 6 ▸(*d*), 6 ▸(*e*) and 6 ▸(*f*) display CXDI views of microspheroids for which the initial concentration of CaCl_2_ was increased to 30 m*M*. For the initial PSS concentration of 8.0 g l^−1^ at [CaCl_2_] = 30 m*M* corresponding to S:Ca = 1.13, the microparticle was small and the shell almost not visible [Fig. 6 ▸(*d*)]. The morphology is quite similar to that obtained with a similar S:Ca ratio at [CaCl_2_] = 8 m*M* [Fig. 6 ▸(*a*)] even if the core/shell structure is slightly less marked. This confirms that both the growth of the microspheres in the early stage and the Ostwald ripening in the later stage are slowed for high initial PSS concentrations. Infrared spectroscopy also highlights two complementary points. First, it reveals that the amount of PSS is released from the microspheroids only when the growth of vaterite crystals occurs [Figs. S9(*a*) and S9(*b*)]. In other words, when the initial concentration of PSS is low, the increase in the average size of the vaterite nanocrystals with reaction time leads to a decrease of the PSS content inside the microspheroids. And when the initial concentration of PSS is high, growth of the vaterite nanocrystals is inhibited and the PSS content inside the microspheroids is high and does not lower with reaction time. This agrees with our previous assumption that growth of CaCO_3_ crystals induces expulsion of a portion of the PSS macromolecules [see Fig. 4 ▸(*b*)]. Second, vaterite is still the only CaCO_3_ polymorph after 1 month of reaction at 70°C [Fig. S9(*c*)]. The phase transformation of vaterite into calcite or aragonite is thus inhibited for a long time.

Now, let us decrease the S:Ca ratio to 0.14 which is quite similar to that of 8 m*M* 0.27 g l^−1^ 24 h displayed in Fig. 6 ▸(*c*), but this time the concentration of CaCl_2_ is 30 m*M*. We observe that the dissolution/recrystallization processes are delocalized over the entire volume of the microparticle leading to, after 72 h of reaction time, a core with macroporous channels which tend to form radial arrangements [Fig. 6 ▸(*e*)]. This ‘hairy’ structure was already reported in previous work (Cherkas *et al.*, 2017 ▸) and may be the result of spherulitic growth (Andreassen, 2005 ▸; Bots *et al.*, 2012 ▸) rather than a process of nanoaggregation. The shell is decorated with hexagonal based bipyramids characteristic of vaterite crystals [see inset of Fig. 6 ▸(*e*)]. To understand why recrystallization takes place both in the core and in the shell, contrary to 8 m*M* 0.27 g l^−1^ 24 h for which the recrystallization is localized in the shell only, microparticles were synthesized with lower reaction times (1 min, 30 min, 2 h, 8 h and 24 h); details are given in Fig. S10. They clearly reveal the presence of a shell already after 1 min reaction time with a thickness between 90 and 150 nm and a core containing mesoporous channels. After 30 min, the core becomes more globally heterogeneous at the mesoscale down to its center and sufficiently porous for the water to penetrate homogeneously into the entire core. After 2 h and more, the pores and the nanoparticles increase in size. This shows that the formation of a core-shell structure in the early stage of the reaction does not necessarily lead to a hollow structure. We propose that the formation of a macroporous core after 72 h for synthesis with [CaCl_2_] = 30 m*M* originates from the early stage of precipitation with the formation of microspheres composed of nanoparticles with a higher size polydispersity than for the synthesis with [CaCl_2_] = 8 m*M*. A homogeneous particle core in the early stage of reaction leads to a ‘homogeneous’ hollow core (*i.e.* all the nanoparticles of the core are dissolved); in contrast, a heterogeneous particle core in the early stage leads to a ‘heterogeneous’ macroporous core (*i.e.* some nanoparticles were dissolved while others grew). The effect of size dispersity of the nanoparticles comprising the initial solid microsphere on the hollowing of the microspheroids is illustrated in Fig. 4 ▸.

Fig. 6 ▸(*f*) part (i) shows a slice of a microspheroid obtained by decreasing the PSS concentration down to 0.27 g l^−1^ with [CaCl_2_] = 30 m*M*. This corresponds to a very low ratio of S:Ca = 0.04. Note that the large microsphere is decorated with a small number of truncated hexagonal base bipyramids and hexagonal prisms of vaterite of micrometre size. An example is shown in Fig. 6 ▸(*f*) part (ii). Again, CXDI reveals that the microspheroid is flattened at the pole and the crystals are aligned such that their *c* axes are mainly oriented along the meridians (Fig. S11).

### 2.10. Oblate and prolate microspheroids

**Figure 7:**
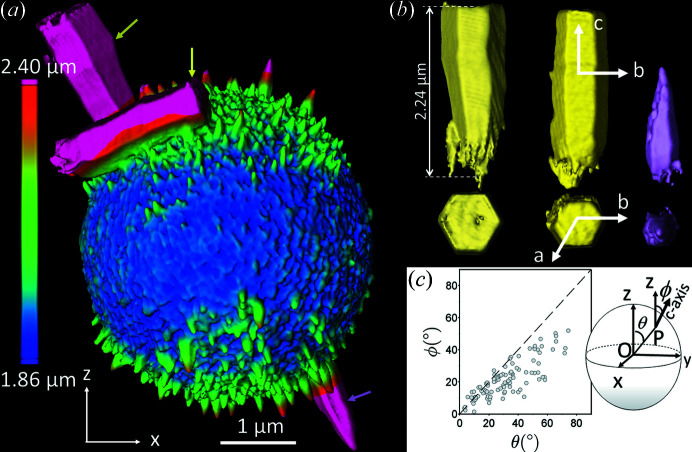
Out-of-plane *c* axis vaterite crystals. CXDI images obtained with *V_x_* = 32.5 nm on a microspheroid from 120 m*M* 1 g g l^−1^ 8 h with (*a*) a 3D view and (*b*) 3D views of the three largest vaterite crystals of the microspheroid shown by the yellow arrows in (*a*). In (*a*), the scale bar corresponds to the radius of the prolate microspheroid. The vectors *a*, *b* and *c* refer here to the crystallographic axes of vaterite according to Kamhi’s model [*P*6_3_/*mmc* space group (Kamhi, 1963 ▸)]. The crystallographic structure was confirmed by TEM (results not shown). Note that one is broken. (*c*) Plot of ϕ as a function of θ for the ∼100 largest nanoparticles of the microspheroid shown in (*a*). The dashed line refers to the equation ϕ = θ. The scheme on the right represents the prolate microspheroid. The center is *O*. The intersection between the microspheroid and a nanoparticle is *P*. θ is the angle between *OP* and the *z* axis. ϕ is the angle of the *c* axis of the nanoparticles and the *z* axis of the microspheroid.

Fig. 7 ▸ displays CXDI views of microspheroids for which the initial concentration of CaCl_2_ was increased to 120 m*M*. Two observations are clearly visible. First, the shape of the spheroid is prolate. This is highlighted in Fig. 7 ▸(*a*) where the colorization refers to the radius of the microspheroid. At the equator, the average radius is close to 1.86 µm, whereas at the pole it is higher (close to 2.02 µm). Second, growth of the outermost crystals is favored at the poles (not at the equator). Some of these crystals exceed 2 µm in length [Fig. 7 ▸(*b*)]. The small vaterite crystals exhibit a hexagonal based pyramidal shape, whereas the largest ones have a hexagonal based prismatic shape. TEM observations confirm that the elongation of the vaterite crystals is along the *c* axis (Fig. S13). Their *c* axes are no longer aligned along the meridians. A careful analysis carried out on more than 100 nanocrystals reveal that the *c* axis is not perfectly radial. The co-latitude ϕ of the *c* axis of the nanoparticles (*i.e.* the angle between the *c* axis and the *z* axis) is slightly lower than the co-latitude θ of the position of the nanoparticles in the microspheroid [Fig. 7 ▸(*c*)]. These findings confirm first that the growth of the nanocrystals during the ripening is favored in regions (pole or equator) where the dipolar energy is minimal and second that the shape of the microspheroid (oblate or prolate) is correlated to the growth of these nanoparticles. We assume that the electrical dipolar energy between the nanoparticles guides the global shape (oblate or prolate) of the spheroids.

**Figure 8:**
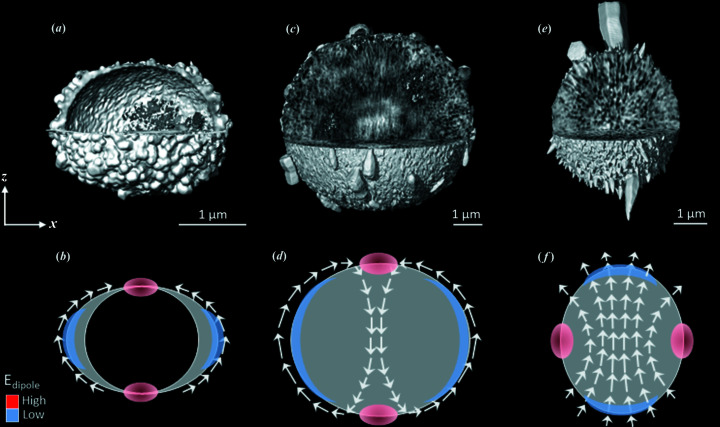
Nanoparticle dipole order controls the shape of the microspheroids. Microspheroids from (*a*) and (*b*) 8 m*M* 1 g l^−1^ 8 h, (*c*) and (*d*) 30 m*M* 0.24 g l^−1^ 24 h, and (*e*) and (*f*) 120 m*M* 1 g l^−1^ 8 h. In (*a*), (*c*) and (*e*) the images were obtained by CXDI for which 1/4 of the microspheroids are cut to reveal the internal structures. In (*b*), (*d*) and (*f*) the schemes represent 2D views of the microspheroids shown in (*a*), (*b*) and (*c*), respectively. White arrows illustrate the *c* axis orientation of the nanocrystals. The red ellipse refers to regions of high dipole energy, *i.e.* where nanocrystal growth does not occurs due to noncollinearity of electrical dipoles (frustrated dipole order). The blue lunules represent regions of low dipole energy, *i.e.* where nanocrystal growth occur due to collinearity of electrical dipoles.

This idea is highlighted in Fig. 8 ▸. The core/shell microspheroid (8 m*M* 1 g l^−1^ 8 h) shown in Fig. 8 ▸(*a*) is oblate because the *c* axes of the nanocrystals of the shell are in the plane of the shell. This is represented in Fig. 8 ▸(*b*) where the white arrows display the orientation of the *c* axis. As we assume that for each nanocrystal an electrical dipole exists along the *c* axis, the co-alignment of the nanocrystals along the meridians leads to a low dipole energy (blue regions close to the equator). On the contrary, at the poles, co-alignment is not possible leading to a high dipole energy (red region) and therefore dissolution of the nanocrystals with time. We propose that this is the in-plane alignment of the nanocrystals which force the pole to dissolve and the microsphere to become oblate. For 30 m*M* 0.24 g l^−1^ 24 h shown in Fig. 8 ▸(*c*), the microspheroid is oblate too with nanocrystal *c* axes aligned along the meridians. The poles are highly porous (see details in Fig. S11) which reveal again that the nanoparticles at the poles dissolve with time. However, in this case, the microspheroid has neither a core/shell structure nor a hollow core. The core displays a fibrous structure oriented approximatively along the *z* direction. Even if the resolution is not fine enough to identify the individual nanocrystals inside the oblate microspheroid, we assume that the fibrous structure reflects their *c* axis orientation. The co-alignment of these dipoles inside and outside the microspheroid is illustrated in Fig. 8 ▸(*d*). As for 8 m*M* 1 g l^−1^ 8 h, the poles and the equator have high and low dipole energies, respectively. Note that the nanocrystals inside the oblate microspheroid cannot grow due to the limited space. For 120 m*M* 1 g l^−1^ 8 h, the fibrous structure inside the microspheroid is also visible along the *z* direction [Fig. 8 ▸(*e*)]. In this case, the poles are regions where crystal growth is favored and the out-of-plane orientation of the *c* axis of the nanocrystals is in the continuity of the fibrous structure. This is shown schematically in Fig. 8 ▸(*f*). The prolate shape of the microspheroid [Fig. 7 ▸(*a*)] can thus be attributed to the fact that crystal growth is favored at the poles [blue regions in Fig. 8 ▸(*f*)] and unfavorable at the equator [red regions in Fig. 8 ▸(*f*)].

## 3. Discussion

CXDI combined and 3D-XRF microscopies allow us to propose a mechanism to explain the self-transformation of solid and polycrystalline vaterite microspheres into core-shell and hollow microspheroids. At the initial reaction time, the nucleation and growth of vaterite led to solid microspheres. pH measurements and optical observations show that the total amount of solid CaCO_3_ formed is delayed in the presence of PSS. A similar effect was observed with polyaspartate and polyacrylate (carboxyl­ate groups) in the precipitation of calcium phosphate (Bigi *et al.*, 2002 ▸), or more prominently with phylic acid [phosphate groups (Xu *et al.*, 2005 ▸)]. The nanoparticles comprising the microsphere are unstable due to their high Gibbs energy. The overgrowth of the nanocrystals located in the outermost layers of the microspheres in contact with the unsaturated solution reduces their Gibbs energy by lowering their specific surface. We propose that the growth of the outermost layers of the microspheres expels PSS macromolecules, some of which are adsorbed by the smaller nanoparticles just beneath, reducing their interfacial free energy. A shell is thus formed with large nanoparticles in its outer part and small nanoparticles in its inner part, both types of nanoparticles being more stable (*i.e.* lower free energy) than those of the core due to either their lower specific surface (outermost layers) or their lower interfacial free energy (innermost layers). Even though XRF could prove that PSS is trapped in the microspheres, the resolution was not sufficient to highlight that the innermost layers of the shell contain more PSS than the outermost layer. The technical progress of synchrotrons and, in particular, the decrease in the size of the nanobeams should make it possible to verify in the coming years the existence of this radial gradient in PSS. The inner part of the shell appears smooth because it is formed from very small nanoparticles (∼Ø 30 nm) which do not grow inwardly because they are kinetically stabilized by PSS. This mechanism may be similar to that encountered in biominerals. For instance, Nassif *et al.* (2005 ▸) observed that the surface of the aragonite crystals comprising abalone nacre is covered with a layer of ACC a few nanometres thick. They proposed, without evidence, that this layer is kinetically stabilized by the expulsion of macromolecules from the adjacent crystallizing aragonite. It is tempting to make a comparison between the shell bilayer architecture of the microspheroids of this article and the complex morphologies encountered in biominerals, sometimes made of well defined crystals on which tightly packed nanosized crystals are present. But what about the crystallographic structure of these nanodomains? In the interlamellar membrane of nacre, nanocrystals of ∼30 nm were found (Macías-Sánchez *et al.*, 2017 ▸). They are surrounded by an amorphous layer 5 to 10 nm thick composed of ACC with organic matter. These partially crystalline domains form the smoothest area of the nacre. The deposition of a thin layer of calcium carbonate in the form of ACC at the surface of crystallized calcium carbonate was encountered also in sea urchin larval spicules (Politi *et al.*, 2008 ▸), in the outer calcitic layer from the pearl oyster *Pinctada margaritifera* (Baronnet *et al.*, 2008 ▸), in the aragonite platelets in nacre from the abalone *Haliotis laevigata* (Nassif *et al.*, 2005 ▸) and in the calcite spines from the sea urchin *Paracentrotus lividus* (Politi *et al.*, 2004 ▸). The stabilization of ACC was also observed in crustaceans and attributed to the presence of low-molecular-weight metabolites (Akiva-Tal *et al.*, 2011 ▸; Sato *et al.*, 2011 ▸). Similarly, PSS also has the ability to stabilize ACC. Smeets *et al.* (2015 ▸) observed by TEM that the nucleation and growth of ACC are favored inside PSS macromolecules. Thus, we cannot exclude the possibility that the nanoparticles of vaterite in the inner part of the shell are covered with a composite layer made of ACC and PSS.

Once the bi-layer shell is formed, the less stable core begins to dissolve. We highlighted that the core is made of an aggregate of small nanoparticles (∼10–30 nm) organized in a dendritic radial structure. Over time, the complete dissolution of the nanoparticles of the core takes place to end with the formation of complete hollow oblate microspheroids. We observed that, for syntheses in which the initial supersaturation state is high ([CaCl_2_] = 30 m*M*), the complete dissolution of the core does not occur. In such cases, the core becomes macroporous with reaction time (not hollow). To link the early and the final stages, let us look at what the literature says in the early stage of the reaction when calcium and carbonate solutions are mixed. SAXS measurements show that the size distribution of primary ACC nanoparticles formed in the first seconds of reaction (*i.e.* before the formation of vaterite) is a function of supersaturation (Bots *et al.*, 2012 ▸; Bolze *et al.*, 2002 ▸, 2004 ▸; Pontoni *et al.*, 2003 ▸; Liu *et al.*, 2010 ▸). By rapid mixing of equimolar solutions of CaCl_2_ and Na_2_CO_3_ with a low initial concentration of 5 m*M*, the size distribution is narrow, and the spherical hydrated ACC particles mainly grow during the first 20 s to a mean value of about 50 nm (Liu *et al.*, 2010 ▸). With a higher initial concentration of [CaCl_2_] = 10 m*M*, the size polydispersity is larger (Bolze *et al.*, 2004 ▸) and the ACC nanoparticles form clusterings or aggregates which may result from the collision between first the primary nanoparticles and then between the clusters. At even higher concentration ([CaCl_2_] = 1 *M*), a gel is formed thst is characteristic of large aggregates (Bots *et al.*, 2012 ▸). The large size polydispersity and the clustering of these primary nanoparticles have also been observed by cryo-TEM from 10 m*M* CaCl_2_ solution (Pichon *et al.*, 2008 ▸). Then, the transformation of ACC into crystalline phases (either calcite or vaterite) takes place by dissolution–recrystallization as observed by *in situ* SAXS (Pontoni *et al.*, 2003 ▸; Bots *et al.*, 2012 ▸), optical (Hu *et al.*, 2012 ▸; Aizenberg *et al.*, 2003 ▸), *in situ* TEM (Nielsen *et al.*, 2014 ▸) and infrared spectroscopy (Shen *et al.*, 2006 ▸) even if the solid-state transformation was also observed by cryo-TEM in very low saturated solutions (Pouget *et al.*, 2009 ▸, 2010 ▸). Gebauer *et al.* (2008 ▸) proposed a link between ACC state and its transformation into calcite or vaterite. They suggested that ACC exists in two states. ACC with weak bonds can transform into vaterite and ACC with stronger bonds is conducive to transformation into calcite. The phase transformation from ACC to vaterite (Nielsen *et al.*, 2014 ▸) or from ACC to calcite (Pontoni *et al.*, 2003 ▸; Kim *et al.*, 2017 ▸) begins at the surface of the ACC particles. Thus, even if the transformation of ACC into vaterite takes place by dissolution–recrystallization, a link exists between these primary ACC nanoparticles and the secondary vaterite structures. It is therefore possible that a lower size polydispersity of the former may have a consequence on that of the latter. We propose that a narrow size distribution of ACC favors a narrow size distribution of vaterite nanocrystals comprising the microspheres and explains (if the PSS concentration is not too high) why the 8 m*M* synthesis leads to hollow microspheroids and the 30 and 120 m*M* syntheses lead to macroporous microspheroids.

In addition to the radial structuring of the shell, piloted by the radial PSS distribution leading to large nanocrystals on the outermost layer and small nanodomains on the innermost layers, a second organization was observed. The *c* axes of the nanocrystals of the shell tend to be aligned with each other. This mechanism may be driven by the dipole–dipole interaction (Xu *et al.*, 2014 ▸; Wang *et al.*, 2015 ▸; Zhu *et al.*, 2009 ▸) and confers to the shell either a oblate (*i.e.* flattened) spheroidal shape with large nanocrystals at the equator and small ones at the poles, or a prolate spheroid shape with large crystals at the pole and small ones at the equator. It is the orientation of the *c* axis of the nanoparticles which determines whether the microspheroids are oblate (*c* axes are oriented in the plane of the shell along the meridians) or prolate (*c* axes are almost radially oriented). In oblate microspheroids, poles can be thus considered as frustrated regions where the nanocrystals cannot be co-aligned. The high dipole energy of the nanoparticles of the poles prevents their growth and finally leads to their complete dissolution. In many chemical systems such as TiO_2_, SrWO_4_, CaCO_3_ (Ye *et al.*, 2010 ▸; Yu *et al.*, 2006 ▸), Fe_2_O_3_ (Cao & Zhu, 2008 ▸), MnO_2_ (Jin *et al.*, 2010 ▸) and Cu_2_O (Sui *et al.*, 2009 ▸), holes on the outer surface of the microspheroid are encountered. We can generalize our observations by proposing that, in the other chemical systems, all these holes are a consequence of the dissolution of nanoparticles in high inter-particle energy regions (not necessary dipole–dipole interactions).

In the low dipole energy regions (poles for prolate microspheroids or the equator for oblate microspheroids), large nanoparticles of vaterite are formed. In somes cases, their length can exceed 2 µm with a hexagonal based bypiramidal or prismatic shape. The synthesis of such vaterite crystals with micrometre size along the *c* axis is rarely reported in the literature, the growth of vaterite usually leads to nanocrystals or microcrystals in the form of platelets (Wang *et al.*, 2015 ▸; Xu *et al.*, 2006 ▸; Dupont *et al.*, 1997 ▸). Our method of synthesis is a simple way to obtain quite large crystals of vaterite, whose crystallographic structure has been under debate up to now (Christy, 2017 ▸). In nature, even though biogenic vaterite is extremely rare, a remarkable example of pyramid-shaped vaterite crystals with a hexagonal base exists in *Herdmania momus* in the form of tunicate spicules (Lowenstam & Abbott, 1975 ▸; Kabalah-Amitai *et al.*, 2013 ▸). Note that these biogenic crystals resemble the synthetic crystals of this study in terms of morphology and hierarchical organization. Our study may thus be useful for developing a deeper understanding of the biomineralization process.

In summary, the self-transformation of solid microspheres into complex oblate and prolate microspheroids was explained using CXDI and 3D-XRF. The stabilizing role of PSS and the minimization of the intercrystal dipolar energy may explain in combination with Ostwald ripening the formation of these sophisticated structures. In addition, the proposed mechanism here for CaCO_3_ may help us to understand the transformations of other chemical systems such as ZnO, TiO_2_, Fe_2_O_3_, Co_3_O_4_, MnO_2_, Cu_2_O, ZnS, CaCO_3_ and Ca_8_H_2_(PO_4_)_6_·5H_2_O for which solid microspheres evolve into core-shell and hollow microspheres/microspheroids.

## 4. Materials and methods

### 4.1. Synthesis of microspheres

Calcium carbonate microspheres were prepared by the precipitation reaction of sodium carbonate with calcium chloride in the presence of sodium poly(4-styrene­sulfonate) (PSS; Aldrich, Mr Mw 70 000). In a typical synthesis, a CaCl_2_ solution of 0.5 *M* (solution 1) and an Na_2_CO_3_ solution of 0.5 *M* (solution 2) are prepared. In addition, several aqueous PSS solutions of 10 ml are prepared. The PSS concentration of these solutions ranges from 0.24 to 8.0 g l^−1^. To prepare an 8 m*M* solution, 0.165 ml of solution 1 is added to a 10 ml aqueous PSS solution. Then, 0.165 ml of solution 2 is added quickly under vigorous stirring. To prepare the 30 m*M* solution, 0.681 ml of solution 1 is added to a 10 ml aqueous PSS solution. Then, 0.681 m*M* of solution 2 is added quickly under vigorous stirring. To prepare the 120 m*M* solution, 4.615 ml of solution 1 is added to a 10 ml aqueous PSS solution. Then, 4.615 m*M* of solution 2 is added quickly under vigorous stirring. The pH of the mixed solution was left unadjusted. The mixture was stirred for 1 min and then placed in an oven and kept under static conditions at 70°C for a period of between 30 min and 3 d. After the reaction, the precipitate was filtered off, washed thoroughly three times with deionized water followed by washing with absolute ethanol before drying in an oven at 60°C for 12 h.

### 4.2. Scanning electron microscopy

Scanning electron microscopy (LEO 1530) was performed using the secondary electron mode of detection and with an accelerating tension of 3 kV. The samples were previously metalized with an ∼5 nm-thick coat of gold. Note that 3D-CXDI analysis was performed before SEM observations so that the microspheroids analyzed by 3D-CXDI were not metalized.

### 4.3. Transmission electron microscopy

Transmission electron microscopy (TEM) studies were carried out (Jeol 2010, Croissy sur Seine, France) with an LaB_6_ thermo-ionic emission source operating at 200 kV. The sample was dispersed in absolute ethanol and one droplet was deposited on a carbon-coated holey film supported by a copper grid before drying.

### 4.4. CXDI measurements and reconstructions

CXDI measurements were performed at the ESRF beamline ID10 (Chushkin *et al.*, 2014 ▸). The X-ray beam produced by a three-undulator source was monochromated by an Si(111) pseudo-channel cut monochromator. Beryllium compound refractive lenses were employed to focus the beam at the sample position. The coherent fraction of the beam was finally selected by roller-blade slits opened to 10 × 10 µm and placed 0.55 m upstream of the sample, giving essentially a plane-wave-like illumination. The sample was mounted on a horizontal ultra-precision rotation stage equipped with *x*, *y*,* z* translations and an on-axis optical microscope. The microscopic samples were deposited on 100 nm-thick Si_3_N_4_ membranes and kept fixed by electrostatic forces. Based on SEM observations, the microparticles imaged by CXDI here are representative of the total microparticle population. 2D diffraction patterns were recorded by a Maxipix detector having 516 × 516 pixels of 55 µm in size. The detector was placed between 1.9 and 5.2 m downstream of the sample and a vacuum flight tube was used to reduce air absorption and scattering. A beamstop was inserted in front of the detector inside the vacuum flight tube to block the intense direct beam and protect the detector from radiation damage. A series of 2D diffraction patterns were recorded at sample tilt angles from about −80° (±5°) to +80° (±5°) to cover a tomographic angular range of about 160° with 0.2–0.5° steps. The measurement time per sample was 2–8 h with 10–25 s per frame. The 2D patterns were assembled into the 3D diffraction volume using linear interpolation and accounting for the Ewald sphere curvature. The real space image reconstruction was achieved by the iterative phase retrieval algorithm applied to the 3D diffraction volume (Chushkin *et al.*, 2014 ▸). The final real space images were obtained by averaging 20 reconstructions. We used 7.0 and 8.1 keV X-rays and different sample-to-detector distances, so the real space images have a voxel size of 32.5 × 32.5 × 32.5 nm for 8 m*M* 1 g l^−1^ 1 min, 8 m*M* 1 g l^−1^ 30 min and 30 m*M* 0.27 g l^−1^ 72 h; 17.7 × 17.7 × 17.7 nm for 30 m*M* 1 g l^−1^ min, 30 m*M* 1 g l^−1^ 30 min, 30 m*M* 1 g l^−1^ 8 h, 30 m*M* 1 g l^−1^ 24 h and 30 m*M* 1 g l^−1^ 72 h; 16.4 × 16.4 × 16.4 nm for 8 m*M* 1 g l^−1^ 8 h, 8 m*M* 1 g l^−1^ 24 h and 8 m*M* 1 g l^−1^ 72 h; 13.8 × 13.8 × 13.8 nm for 8 m*M* 1.94 g l^−1^ 24 h; and 12.6 × 12.6 × 12.6 nm for 8 m*M* 0.27 g l^−1^ 24 h. The reconstructed real space images are subject to smooth, low-frequency density variations due to missing data behind the beamstop, strongly resembling the ‘unconstrained modes’ reported by Thibault *et al.* (2006 ▸). To remedy these density variations, a simple spatial flattening of the electron density was applied to the reconstructions by subtracting in real space a 3D Gaussian function centered at the mass center (Cherkas *et al.*, 2017 ▸; Beuvier *et al.*, 2019 ▸). After subtracting the 3D Gaussian function, voxels with negative density values were set to zero. *VG Studio* Max (https://www.volumegraphics.com/en/products/vgsm/ct-reconstruction-data-quality-analysis.html) was used for the visualization of the surface of the 3D volume and the 2D slices obtained by CXDI. The sphere method was used to determine the shell thicknesses.

### 4.5. X-ray nanofluorescence at ID16

The XRF tomography experiments were performed at the ID16A nano-imaging beamline of the European Synchrotron Radiation Facility (ESRF), France (Da Silva *et al.*, 2017 ▸). The beamline was set to provide an X-ray nanofocus of 40 nm (H) × 42 nm (V) with about 10^9^ photons s^−1^ with energy an of 17.05 keV (bandwidth of Δ*E*/*E* ≃ 10^−2^). The nanofocus size was estimated through knife-edge scans on an Au-made lithographic pattern developed by Minatec (Grenoble, France). Two beam attenuators, one made from 5 µm Au and the other from 2 mm Si, were inserted in the beam path. The sample was attached at the top of a silica tip of around 5–10 µm size and was inserted in a high-vacuum chamber (∼10^−7^ mbar) at the beamline for the XRF analyses. We observed that, as soon as the beam passed through the sample, the core of the microparticle vibrated and broke the shell. This problem was resolved by metalizing the sample with a (conductive) carbon coating 5–10 nm thick. The metallization evacuates the charges. X-ray tomography was performed by acquiring 80 2D fluorescence maps at different angular positions of the sample over 180°, with a 2.25° angular step size. The sample was scanned on-the-fly through the X-ray nanofocus, with the X-ray fluorescence emission collected by a pair of six-element silicon drift detectors (SDDs), manufactured by SGX Sensortech, positioned perpendicular to the beam path on each side of the sample. All SDD elements are composed of a 450 µm-thick Si element fitted with a Be window (25 µm in thickness) and a homemade collimator resulting in a 6.5 cm^2^ total active area. A shielding plate with a pinhole is attached to the detector to prevent unwanted scattered X-ray photons towards the detector elements, particularly X-ray fluorescent emission from the focusing optics and surroundings. A diode is installed behind the sample to monitor the flux while continuously scanning the sample. The field of view of each 2D XRF map was 7 µm (H) × 5 µm (V), scanned with a step size of 65 nm, which yield 2D images with a pixel size of 65 nm. The acquisition time per scan point was 700 ms. The XRF tomography data processing includes several steps. The raw fluorescence spectra, summed over the different detector elements, were fitted using the *PyMCA* software (Solé *et al.*, 2007 ▸) to obtain 2D XRF maps of the different elements following the steps published by Yang *et al.* (2019 ▸). Then, we used the 2D XRF maps of Ca for the image alignment using tomographic consistency methods as described by Guizar-Sicairos *et al.* (2011 ▸). The aligned XRF maps for Ca and S were then cast into the filtered back projection algorithm for the tomographic reconstruction using *Toupy*. We used a Hanning filter and a frequency cutoff of 0.9 in the reconstruction. The resulting 3D images had a tomographic volume of 7 × 5 × 5 µm and an isotropic voxel size of 65 nm.

### 4.6. Infrared spectroscopy

All spectra were recorded from 400 to 4000 cm^−1^ using the Alpha Bruker 24FT-IR spectrometer. Each spectrum was an average of 200 scans using a spectral resolution of 2 cm^−1^. For each sample, a small amount of powder was placed on the diamond crystal and pressed slightly. For the experiment shown in Fig. S12 of the supporting information, a drop was placed on the diamond crystal without pressing.

## 5. Related literature

The following references are cited in the supporting information: Farhadi Khouzani *et al.* (2015 ▸); Xu & Poduska (2014 ▸); Beuvier *et al.* (2013 ▸); Vagenas *et al.* (2003 ▸); Rodriguez-Blanco *et al.* (2011 ▸).

## Supplementary Material

Supporting information and figures. DOI: 10.1107/S2052252522006108/lq5044sup1.pdf

## References

[bb1] Aizenberg, J., Muller, D. A., Grazul, J. L. & Hamann, D. R. (2003). *Science*, **299**, 1205–1208. 10.1126/science.1079204 12595685

[bb2] Akiva-Tal, A., Kababya, S., Balazs, Y. S., Glazer, L., Berman, A., Sagi, A. & Schmidt, A. (2011). *Proc. Natl Acad. Sci. USA*, **108**, 14763–14768. 10.1073/pnas.1102608108 PMC3169114 21873244

[bb3] Andreassen, J.-P. (2005). *J. Cryst. Growth*, **274**, 256–264.

[bb4] Baronnet, A., Cuif, J. P., Dauphin, Y., Farre, B. & Nouet, J. (2008). *Miner. Mag.* **72**, 617–626.

[bb103] Beuvier, T., Bardeau, J., Calvignac, B., Corbel, G., Hindré, F., Grenèche, J., Boury, F. & Gibaud, A. (2013). *J. Raman Spectrosc.* **44**, 489–495.

[bb5] Beuvier, T., Probert, I., Beaufort, L., Suchéras-Marx, B., Chushkin, Y., Zontone, F. & Gibaud, A. (2019). *Nat. Commun.* **10**, 751. 10.1038/s41467-019-08635-x PMC6375944 30765698

[bb6] Bigi, A., Boanini, E., Walsh, D. & Mann, S. (2002). *Angew. Chem. Int. Ed.* **41**, 2163–2166. 19746631

[bb7] Bolze, J., Peng, B., Dingenouts, N., Panine, P., Narayanan, T. & Ballauff, M. (2002). *Langmuir*, **18**, 8364–8369.

[bb8] Bolze, J., Pontoni, D., Ballauff, M., Narayanan, T. & Cölfen, H. (2004). *J. Colloid Interface Sci.* **277**, 84–94. 10.1016/j.jcis.2004.04.029 15276042

[bb9] Bots, P., Benning, L. G., Rodriguez-Blanco, J.-D., Roncal-Herrero, T. & Shaw, S. (2012). *Cryst. Growth Des.* **12**, 3806–3814.

[bb10] Cao, S.-W. & Zhu, Y.-J. (2008). *J. Phys. Chem. C*, **112**, 6253–6257.

[bb11] Cherkas, O., Beuvier, T., Breiby, D. W., Chushkin, Y., Zontone, F. & Gibaud, A. (2017). *Cryst. Growth Des.* **17**, 4183–4188.

[bb12] Cherkas, O., Beuvier, T., Zontone, F., Chushkin, Y., Demoulin, L., Rousseau, A. & Gibaud, A. (2018). *Adv. Powder Technol.* **29**, 2872–2880.

[bb13] Christy, A. G. (2017). *Cryst. Growth Des.* **17**, 3567–3578.

[bb14] Chushkin, Y., Zontone, F., Lima, E., De Caro, L., Guardia, P., Manna, L. & Giannini, C. (2014). *J. Synchrotron Rad.* **21**, 594–599. 10.1107/S1600577514003440 24763650

[bb15] Da Silva, J. C., Pacureanu, A., Yang, Y., Bohic, S., Morawe, C., Barrett, R. & Cloetens, P. (2017). *Optica*, **4**, 492–495.

[bb16] Delmas, T., Piraux, H., Couffin, A.-C., Texier, I., Vinet, F., Poulin, P., Cates, M. E. & Bibette, J. (2011). *Langmuir*, **27**, 1683–1692. 10.1021/la104221q 21226496

[bb17] Du, Q., Tang, K., Marioara, C. D., Andersen, S. J., Holmedal, B. & Holmestad, R. (2017). *Acta Mater.* **122**, 178–186.

[bb18] Dupont, L., Portemer, F. & Figlarz, M. (1997). *J. Mater. Chem.* **7**, 797–800.

[bb19] Fang, Y., Xia, Z., Yu, F., Sha, J., Wang, Y. & Zhou, W. (2012). *CrystEngComm*, **14**, 8615–8619.

[bb101] Farhadi Khouzani, M., Chevrier, D. M., Güttlein, P., Hauser, K., Zhang, P., Hedin, N. & Gebauer, D. (2015). *CrystEngComm*, **17**, 4842–4849.

[bb20] Gao, Y.-X., Yu, S.-H., Cong, H., Jiang, J., Xu, A.-W., Dong, W. F. & Cölfen, H. (2006). *J. Phys. Chem. B*, **110**, 6432–6436. 10.1021/jp0606199 16570935

[bb21] Gebauer, D., Völkel, A. & Cölfen, H. (2008). *Science*, **322**, 1819–1822. 10.1126/science.1164271 19095936

[bb22] Gehrke, N., Cölfen, H., Pinna, N., Antonietti, M. & Nassif, N. (2005). *Cryst. Growth Des.* **5**, 1317–1319.

[bb23] Guizar-Sicairos, M., Diaz, A., Holler, M., Lucas, M. S., Menzel, A., Wepf, R. A. & Bunk, O. (2011). *Opt. Express*, **19**, 21345–21357. 10.1364/OE.19.021345 22108985

[bb24] Guo, E., Zeng, G., Kazantsev, D., Rockett, P., Bent, J., Kirkland, M., Van Dalen, G., Eastwood, D. S., StJohn, D. & Lee, P. D. (2017). *RSC Adv.* **7**, 15561–15573.

[bb25] Hu, Q., Nielsen, M. H., Freeman, C. L., Hamm, L. M., Tao, J., Lee, J. R. I., Han, T. Y. J., Becker, U., Harding, J. H., Dove, P. M. & De Yoreo, J. J. (2012). *Faraday Discuss.* **159**, 509–523.

[bb26] Jin, L., Xu, L., Morein, C., Chen, C., Lai, M., Dharmarathna, S., Dobley, A. & Suib, S. L. (2010). *Adv. Funct. Mater.* **20**, 3373–3382.

[bb27] Kabalah-Amitai, L., Mayzel, B., Kauffmann, Y., Fitch, A. N., Bloch, L., Gilbert, P. U. P. A. & Pokroy, B. (2013). *Science*, **340**, 454–457. 10.1126/science.1232139 23620047

[bb28] Kamhi, S. R. (1963). *Acta Cryst.* **16**, 770–772.

[bb29] Kim, Y.-Y., Freeman, C. L., Gong, X., Levenstein, M. A., Wang, Y., Kulak, A., Anduix-Canto, C., Lee, P. A., Li, S., Chen, L., Christenson, H. K. & Meldrum, F. C. (2017). *Angew. Chem. Int. Ed.* **56**, 11885–11890. 10.1002/anie.201706800 PMC5638089 28767197

[bb30] Liu, B. & Zeng, H. C. (2005). *Small*, **1**, 566–571. 10.1002/smll.200500020 17193487

[bb31] Liu, J., Pancera, S., Boyko, V., Shukla, A., Narayanan, T. & Huber, K. (2010). *Langmuir*, **26**, 17405–17412. 10.1021/la101888c 20961060

[bb32] Lowenstam, H. A. & Abbott, D. P. (1975). *Science*, **188**, 363–365. 10.1126/science.1118730 1118730

[bb33] Macías-Sánchez, E., Willinger, M. G., Pina, C. M. & Checa, A. G. (2017). *Sci. Rep.* **7**, 12728. 10.1038/s41598-017-12673-0 PMC5629257 28983081

[bb34] Nassif, N., Pinna, N., Gehrke, N., Antonietti, M., Jäger, C. & Cölfen, H. (2005). *Proc. Natl Acad. Sci USA*, **102**, 12653–12655. 10.1073/pnas.0502577102 PMC1200266 16129830

[bb35] Nielsen, M. H., Aloni, S. & De Yoreo, J. J. (2014). *Science*, **345**, 1158–1162. 10.1126/science.1254051 25190792

[bb36] Ostwald, W. (1897). *Z. Phys. Chem.*, **22U**, 289.

[bb37] Pichon, B. P., Bomans, P. H. H., Frederik, P. M. & Sommerdijk, N. A. J. M. (2008). *J. Am. Chem. Soc.* **130**, 4034–4040. 10.1021/ja710416h 18303894

[bb38] Politi, Y., Arad, T., Klein, E., Weiner, S. & Addadi, L. (2004). *Science*, **306**, 1161–1164. 10.1126/science.1102289 15539597

[bb39] Politi, Y., Metzler, R. A., Abrecht, M., Gilbert, B., Wilt, F. H., Sagi, I., Addadi, L., Weiner, S., Gilbert, P. U. P. A. & Gilbert, P. (2008). *Proc. Natl Acad. Sci. USA*, **105**, 17362–17366. 10.1073/pnas.0806604105 PMC2582271 18987314

[bb40] Pontoni, D., Bolze, J., Dingenouts, N., Narayanan, T. & Ballauff, M. (2003). *J. Phys. Chem. B*, **107**, 5123–5125.

[bb41] Pouget, E. M., Bomans, P. H. H., Dey, A., Frederik, P. M., de With, G. & Sommerdijk, N. A. J. M. (2010). *J. Am. Chem. Soc.* **132**, 11560–11565. 10.1021/ja102439r 20669942

[bb42] Pouget, E. M., Bomans, P. H. H., Goos, J. A. C. M., Frederik, P. M., de With, G. & Sommerdijk, N. A. J. M. (2009). *Science*, **323**, 1455–1458. 10.1126/science.1169434 19286549

[bb105] Rodriguez-Blanco, J. D., Shaw, S. & Benning, L. G. (2011). *Nanoscale* **3**, 265–271. 10.1039/c0nr00589d 21069231

[bb43] Sato, A., Nagasaka, S., Furihata, K., Nagata, S., Arai, I., Saruwatari, K., Kogure, T., Sakuda, S. & Nagasawa, H. (2011). *Nat. Chem. Biol.* **7**, 197–199. 10.1038/nchembio.532 21336282

[bb44] Shen, Q., Wei, H., Zhou, Y., Huang, Y., Yang, H., Wang, D. & Xu, D. (2006). *J. Phys. Chem. B*, **110**, 2994–3000. 10.1021/jp055063o 16494300

[bb45] Smeets, P. J. M., Cho, K. R., Kempen, R. G. E., Sommerdijk, N. A. J. M. & De Yoreo, J. J. (2015). *Nat. Mater.* **14**, 394–399. 10.1038/nmat4193 25622001

[bb100] Solé, V. A., Papillon, E., Cotte, M., Walter, P. & Susini, J. (2007). *At. Spectrosc.* **62**, 63–68.

[bb46] Sui, Y., Zhang, Y., Fu, W., Yang, H., Zhao, Q., Sun, P., Ma, D., Yuan, M., Li, Y. & Zou, G. (2009). *J. Cryst. Growth*, **311**, 2285–2290.

[bb47] Thibault, P., Elser, V., Jacobsen, C., Shapiro, D. & Sayre, D. (2006). *Acta Cryst.* A**62**, 248–261. 10.1107/S0108767306016515 16788265

[bb48] Ummadisingu, A. & Grätzel, M. (2018). *Sci. Adv.* **4**, e1701402. 10.1126/sciadv.1701402 PMC5804582 29423441

[bb104] Vagenas, N. V., Gatsouli, A. & Kontoyannis, C. G. (2003). *Talanta*, **59**, 831–836. 10.1016/S0039-9140(02)00638-0 18968970

[bb49] Wang, Y.-Y., Yao, Q.-Z., Li, H., Zhou, G.-T. & Sheng, Y.-M. (2015). *Cryst. Growth Des.* **15**, 1714–1725.

[bb50] Xu, A. W., Antonietti, M., Cölfen, H. & Fang, Y. P. (2006). *Adv. Funct. Mater.* **16**, 903–908.

[bb51] Xu, A. W., Yu, Q., Dong, W. F., Antonietti, M. & Cölfen, H. (2005). *Adv. Mater.* **17**, 2217–2221.

[bb102] Xu, B. & Poduska, K. M. (2014). *Phys. Chem. Chem. Phys.* **16**, 17634–17639. 10.1039/c4cp01772b 25027312

[bb52] Xu, J.-S. & Zhu, Y.-J. (2011). *CrystEngComm*, **13**, 5162–5169.

[bb53] Xu, Y., Ma, G. & Wang, M. (2014). *Cryst. Growth Des.* **14**, 6166–6171.

[bb54] Yang, Y., Fus, F., Pacureanu, A., da Silva, J. C., De Nolf, W., Biot, C., Bohic, S. & Cloetens, P. (2019). *Anal. Chem.* **91**, 6549–6554. 10.1021/acs.analchem.8b05957 31026149

[bb55] Ye, M., Chen, Z., Wang, W., Shen, J. & Ma, J. (2010). *J. Hazard. Mater.* **184**, 612–619. 10.1016/j.jhazmat.2010.08.080 20855164

[bb56] Yu, J. G., Guo, H. T., Davis, S. A. & Mann, S. (2006). *Adv. Funct. Mater.* **16**, 2035–2041.

[bb57] Yu, S.-H., Cölfen, H. & Antonietti, M. (2003). *J. Phys. Chem. B*, **107**, 7396–7405. 10.1021/jp034009+ 26312744

[bb58] Zhu, Y., Liu, Y., Ruan, Q., Zeng, Y., Xiao, J., Liu, Z., Cheng, L., Xu, F. & Zhang, L. (2009). *J. Phys. Chem. C*, **113**, 6584–6588.

