# Supplementary material for: Self-transformation of solid CaCO3 microspheres into core-shell and hollow hierarchical structures revealed by coherent X-ray diffraction imaging
Source: IUCrJ. 2022 Jul 16;9(Pt 5):580–93. doi: 10.1107/S2052252522006108 (PMC9438498; doi:10.1107/S2052252522006108)
Supplement: Supplementary file 1 [file m-09-00580-sup1.pdf]

IUCrJ

Volume 9 (2022)

Supporting information for article:

Self-transformation of solid CaCO3 microspheres into core-shell and hollow hierarchical structures revealed by coherent X-ray diffraction tomography

Thomas Beuvier, Yuriy Chushkin, Federico Zontone, Alain Gibaud, Oxana Cherkas, Julio Da Silva and Irina Snigireva

Summary

TEM images of *8mM-1gL-24h* and *8mM-1gL-72h* microspheroids. 2

In situ infrared spectroscopy. 2

TEM images of microspheroids of vaterite *8mM-1gL-2h*. 4

Orientation of platelets along the meridians of the spheroids. 5

Two other microspheroids *8mM-1gL-8h* observed by CXDI. 6

SEM and TEM images of the vaterite microspheroids *8mM-1gL-8h* 8

pH measurements and optical images show that PSS slows down the precipitation of $CaCO_3$. 9

SEM images of *8mM-0.27gL-8h*. 11

Infrared spectroscopy shows a correlation between the growth of $CaCO_3$ and the release of PSS macromolecules. 12

CXDI of *30mM-1gL* 14

The microspheroid *30mM-0.24gL-24h* is flattened at the poles 15

2D thick slice allows better observation of the bilayered structure of the shell 17

Electron diffraction confirms the c-axis alignment of the nanocrystals 18

TEM images of *8mM-1gL-24h* and *8mM-1gL-72h* microspheroids.

TEM images of *8mM-1gL-24h* and *8mM-1gL-72h* microspheroids are shown in figures S1. The microspheroids are oriented such that the polar axis is roughly perpendicular to the TEM grid. It is thus possible to observe the nanoparticles of the poles. Their diameters are between 30 and 50 nm.

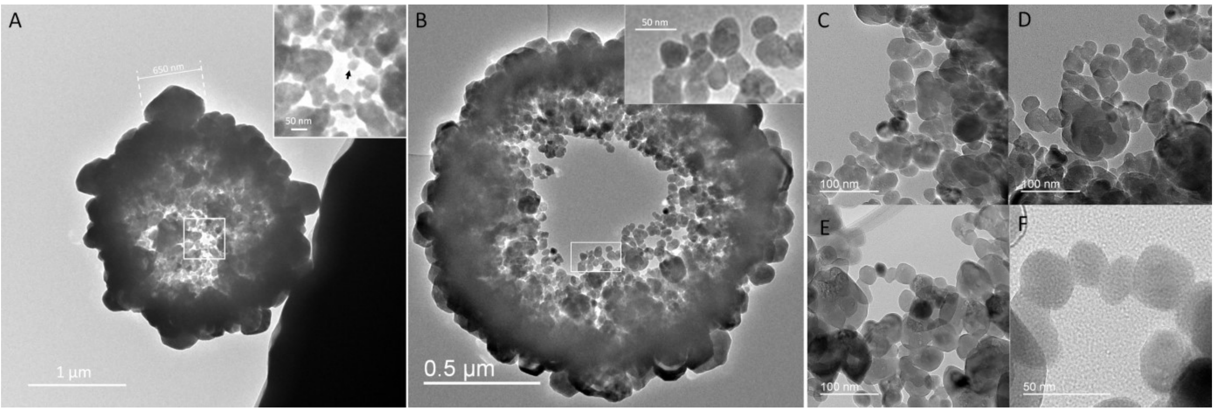

Figure S1: TEM images on *8mM-1gL-24h* and *8mM-1gL-72h* microspheroids. (A) *8mM-1gL-24h.* In the full image, a vaterite crystal of 650nm width is observed. It is located close to the equator. In the inset, a zoom of the pole region is marked by a white square in the full image. The black arrow shows a nanoparticle of 30nm. (B,C) *8mM-1gL-72h*. In the inset of (B), a zoom of the pole region is marked by a white rectangle in the full image. The size of the nanoparticles of the pole is between 30 and 50 nm. In (C), TEM images of the nanoparticles present at the poles of microspheroids of vaterite *8mM-1gL-72h*. The size of the nanoparticles of the pole is between 30 and 50 nm.

In situ infrared spectroscopy.

What about the zone separating the shell from the core in the early stages? Was it, before dissolution, composed of vaterite nanocrystals which then dissolved by Ostwald ripening (vaterite to vaterite transformation) or was it composed of amorphous nanodomains? It is indeed questionable whether the interior of the microspheres is crystalline or amorphous and whether the dissolution/recrystallisation takes place with phase change (ACC to vaterite) or without phase change (vaterite to vaterite transformation by Ostwald ripening). The ex-situ measurements showing that after drying the microspheres consist of vaterite (no ACC) do not allow to conclude on the presence or not of ACC during the formation of the microspheres. To detect the presence or absence of amorphous calcium carbonate, in-situ experiments should be carried out. We undertook in-situ measurements by infrared spectroscopy. Upon injection of the Na_2CO_3 solution into the $CaCl_2$-PSS solution (*8mM-1gL synthesis*), a drop of the suspension was taken very quickly (after ~30s of reaction), and placed on the diamond surface of the infrared spectrometer. Infrared measurements were taken every 30 seconds. The results are summarized in the figure below:

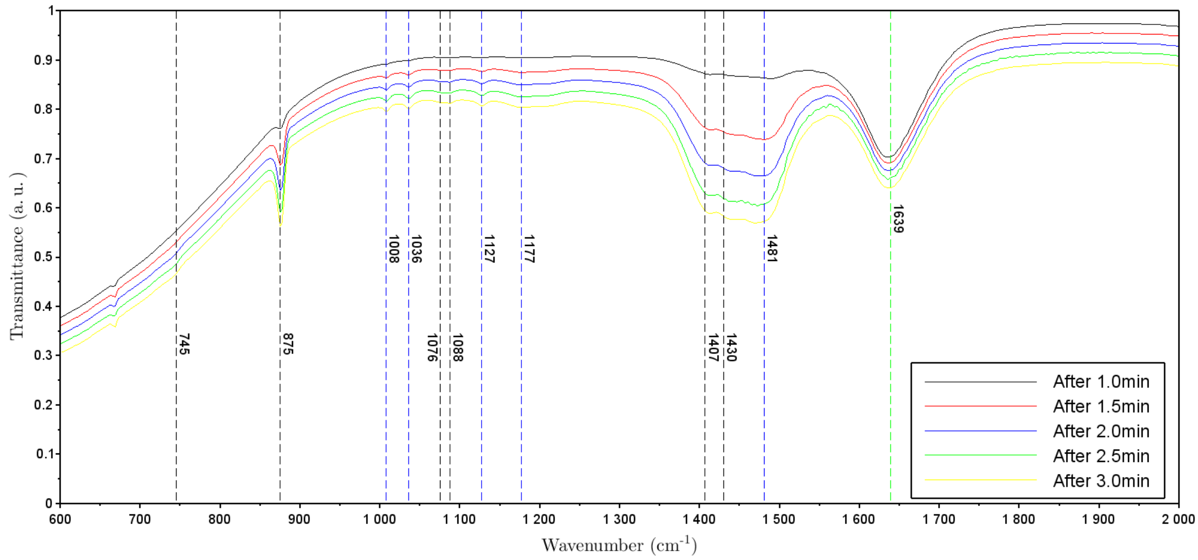

Figure S2: In-situ infrared spectroscopy. Infrared spectra in the range 600-2000 cm^{-1} of a drop of the *8mM-1gL* suspension collected 30 seconds after the beginning of the reaction.

The above spectra can be explained as follows:

- vaterite with the following peaks:
  - at 875 cm^{-1} (ν_2 mode),
  - at 1076 cm^{-1} and 1088 cm^{-1} (ν_1 mode),
  - between 1350 cm^{-1} and 1550 cm^{-1} (ν_3 mode).

Note that the ν_4 mode at 745 cm^{-1} has a very low intensity (see for comparison figure S9 on dry powder). This mode is not visible here.

- PSS with the following peaks (see for comparison figure S9) :
  - at 1008 cm^{-1},
  - at 1036 cm^{-1},
  - at 1127 cm^{-1},
  - at 1177 cm^{-1},
  - at 1481 cm^{-1}.
- Water with the following peak:
  - at 1639 cm^{-1} (δH_2O).

These measurements show that the vaterite variety forms as early as the first minute. The amount of vaterite detected increases with the duration of the reaction probably largely due to the sedimentation of the vaterite particles on the diamond surface.

According to literature,[1,2] ACC has the characteristic peaks:

  - at 863 cm^{-1} (ν_2 mode),

- at 1073 cm^{-1} (ν_1 mode),
- A broad band between 1350 and 1550 (ν_3 mode).

The ν_1 and ν_2 modes of ACC were not detected here. In our in-situ experiments, note that the penetration depth of the IR evanescent wave is calculated to be close to 2µm. Thus, if ACC were present, we would be observed by IR – ATR. This result supports the idea that in the early stage of reaction, the solid microspheres are mainly composed of nanocrystals of vaterite which evolve with time by Ostwald ripening.

[1] Farhadi-Khouzani, M., et al. Disordered amorphous calcium carbonate from direct precipitation. CrystEngComm 17, 4842-4849 (2015)
[2] Shen, Q., et al. Properties of Amorphous Calcium Carbonate and the Template Action of Vaterite Spheres J. Phys. Chem. B 110, No. 7, (2006)

TEM images of microspheroids of vaterite *8mM-1gL-2h*.

After 2h of reaction, the core-shell structure is observed (Figure S3A). Some microspheroids are broken in such a way that the nanoparticles of the core can be observed without being hindered by the shell particles (Figure S3B). The width of the nanoparticles of the core is between 10 and 25 nm. These values are smaller than the one of the outer part of the shell (Figure S3C). The width of the nanoparticles of the shell is between 80 and 100 nm.

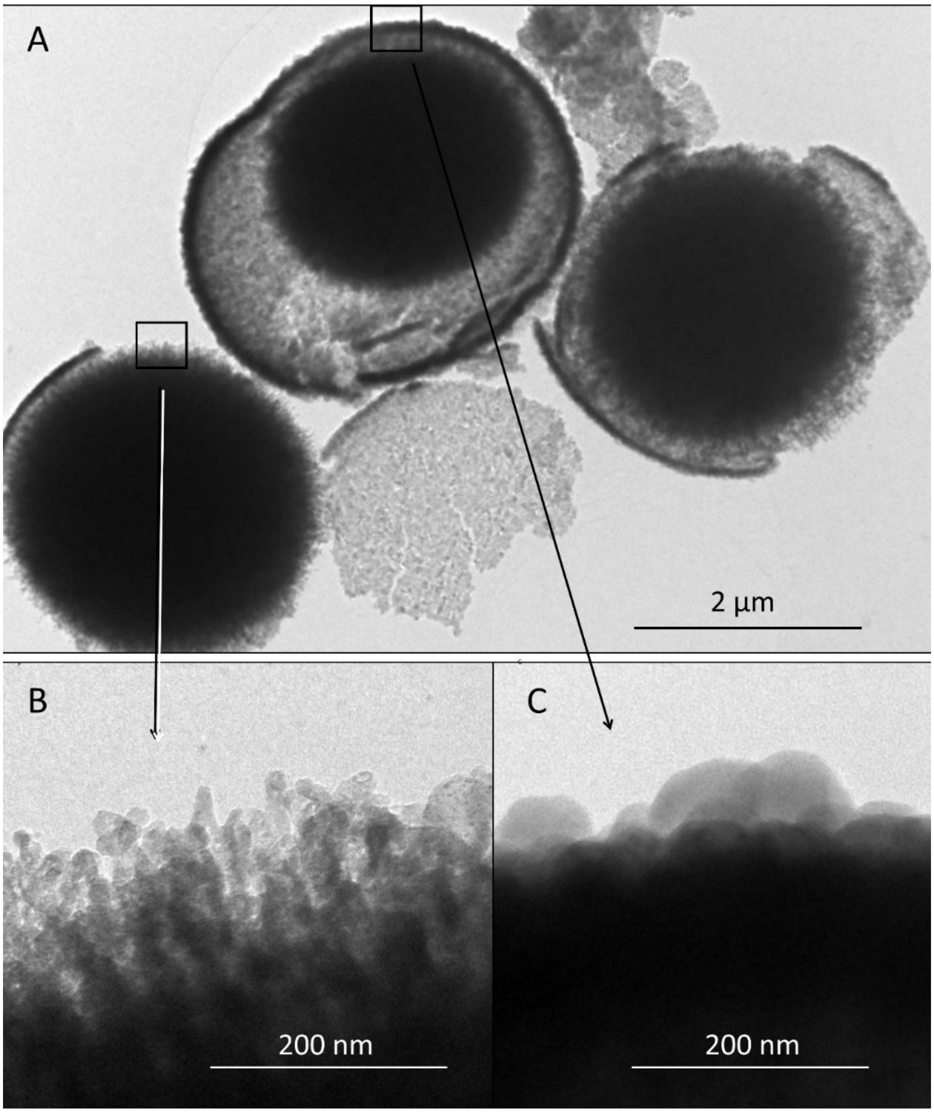

Figure S3: TEM images of microspheroids of vaterite *8mM-1gL-2h*. (A) Large view showing 3 microspheroids. The black arrow shows a broken shell. The squares refer to the areas magnified in B and C. (B) The exterior of the core. The width of the nanoparticles is between 10 and 25 nm. (C) The exterior of the shell. The width of the nanoparticles is between 80 and 100 nm.

Orientation of platelets along the meridians of the spheroids.

Figure S4(A) shows vaterite crystals extracted from the surface of the *8mM-1gL-8h* microspheroid observed in figure 3(E-H). In Figure S4(B), this microspheroid is traced in semi-transparency with the 6 crystals shown in Figure S4(A). Each of these crystals is associated with a white arrow pointing in crystallographic direction c according to the Kahmi model.[3] These arrows make it possible to highlight the orientation of the crystals along the meridians of the microspheroid. Figure S4(C) shows a schematic representation of a vaterite crystal with a truncated bipyramidal morphology. In addition, the crystallographic axes *a*, *b*, *c* of the vaterite according to the Kahmi model were added.

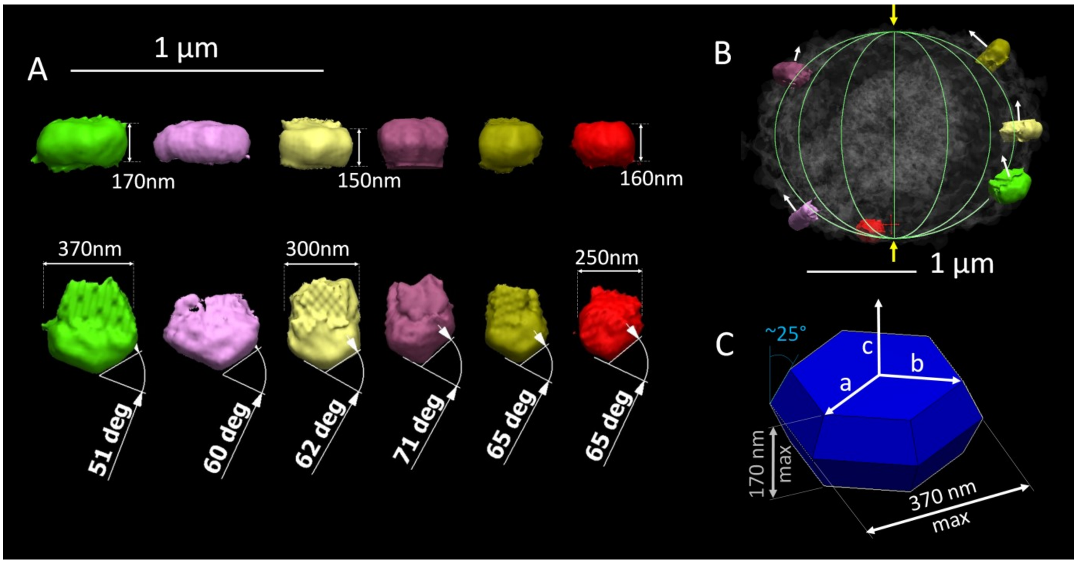

Figure S4: Orientation of platelets along the meridians of the spheroids. CXDI on the *8mM-1gL-8h* microspheroid with (A) 6 nanocrystals extracted from the microspheroid, (B) the microspheroid viewed in semi-transparency except for the 6 nanocrystals for which no transparency was applied, (C) a schematic representation of a nanocrystal of vaterite with the truncated bipyramidal morphology. In B, the white arrows represent the c-axis of vaterite according to the Kahmi model. The yellow arrows represents the poles of the microspheroid.

Two other microspheroids *8mM-1gL-8h* observed by CXDI.

The figures S5 show two other microspheroids *8mM-1gL-8h* observed by CXDI with a resolution of 16.4 nm (Figure S5(B-F) and 32.5 nm (Figure S5(H-L)). For each figure, an SEM image of a microspheroid is shown in A and G. In B and H, two 3D CXDI views of the same microspheroids allow the comparison with the SEM images. The two microspheroids are reoriented to be viewed along the *xz*-plan with a (C,I) 3D view and a (D,J) 2D view and along the *xy*-plan with a (E,K) 3D view and a (F,L) 2D view. These figures show 4 important points. First, it shows that the images obtained by nanotomography are similar to those obtained by SEM, confirming that the CXDI reconstructions are correct (Figures S5(A,B,G,H)). Secondly, the CXDI results confirm that the shell is thicker at the equator than at the pole (Figures S5(D,J,F,L)). Thirdly, by approximating the inner part of the shell to an oblate spheroid, it is possible to extract the main major axis A and the main minor axis C and thus calculate their eccentricity $e = (A^2\text{-}C^2)^{0.5}/A$. The values obtained are very similar to each other. The minor axis is equal to C = 1.84 µm for the particle shown in Figure S5D and C = 1.85 µm for the one shown in figure S5J. The major axis is between A = 2.17 µm (Figure S5F) and A = 2.16 µm (Figure S5L). This leads to eccentricities of e = 0.53 and e = 0.52, respectively. Finally, the core is not totally dissolved. The three microspheroids *8mM-1gL-8h* observed by CXDI (the one shown in figure 3(E-H), and the 2 others in figures S5) are at the same aging point. In other words, aging may take place in the same way for all the particles.

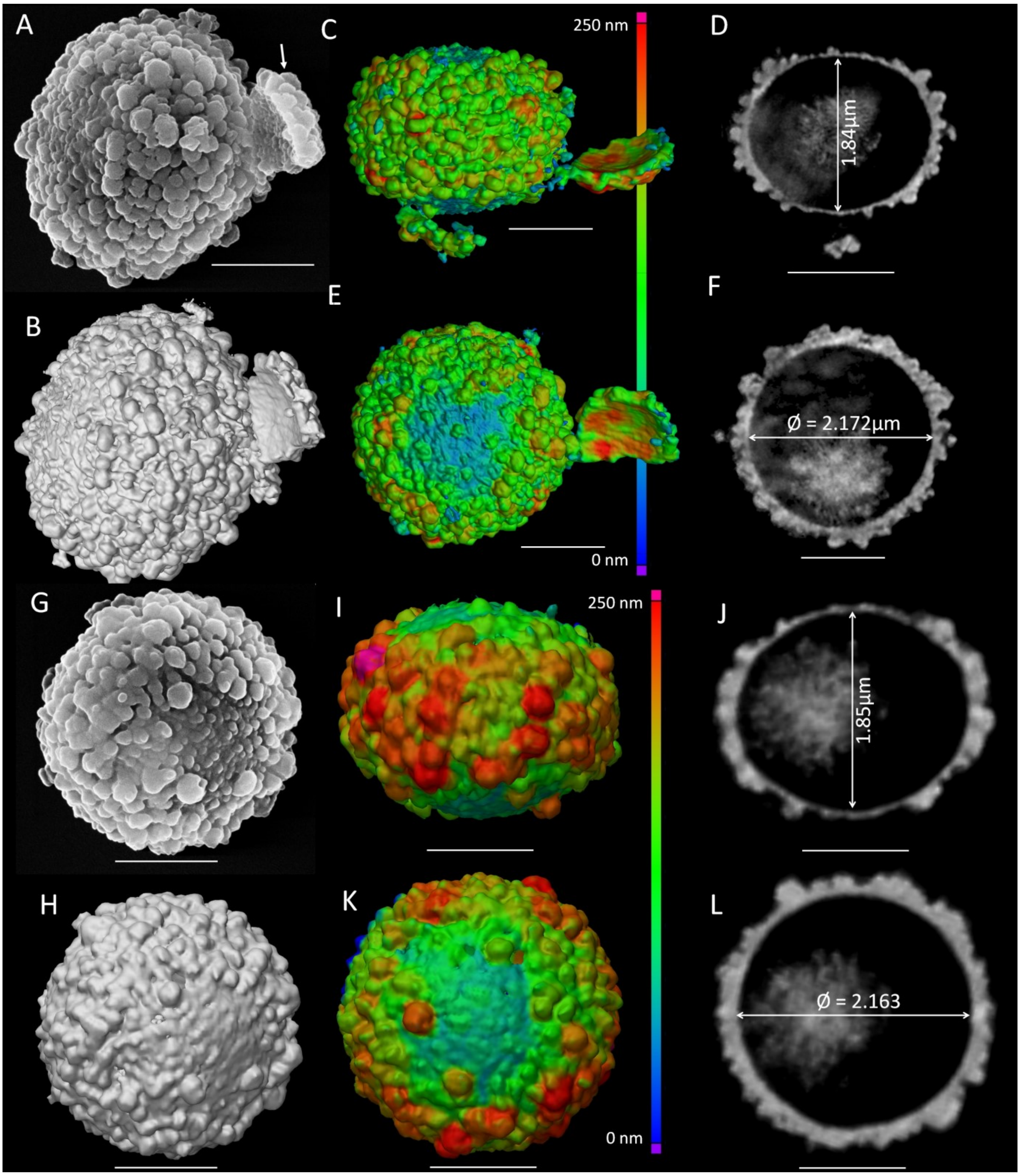

Figure S5: Two other microspheroids 8mM-1gL-8h observed by CXDI. (A,G) SEM images of two microspheroids *8mM-1gL-8h*, (B,H) 3D CXDI views of the same microspheroids with a voxel size of (B) 16.4 and (H) 32.5 nm, (C, D, I, J) 3D and 2D views of the same microspheroids viewed along the xz-plan, (E, F,K,L) 3D and 2D views of the same microspheroids, viewed along the xy-plan. The colorization on (C), (E), (I) and (K) refers to the thickness of the shell. The average shell thickness is equal to 178 and 185 nm for the first and the second microspheroids, respectively. In (A), the arrow shows that some microspheroids are broken.

SEM and TEM images of the vaterite microspheroids *8mM-1gL-8h*.

Figure S6A shows an SEM image of the vaterite *8mM-1gL-8h* microspheroids. The microspheroids display roughly the same size. Various broken particles are observed. In the TEM images shown in Figure S6B-S6D, most of the microspheroids have a core that is not totally dissolved as expected from CXDI results. Some microspheroids have lost their core probably because their shell broke during the preparation of the grid. In figure S6D, the microspheroid is oriented so that the axis of the poles is parallel to the grid. This orientation allows us to confirm that the nanocrystals of the poles located on the outer part of the shell are less developed than those of the equator. In addition, the internal diameter of the main minor axis is close to C=1.81µm. This value is similar to those measured on the microspheroids presented in the figures 3G, S5D, and S5J.

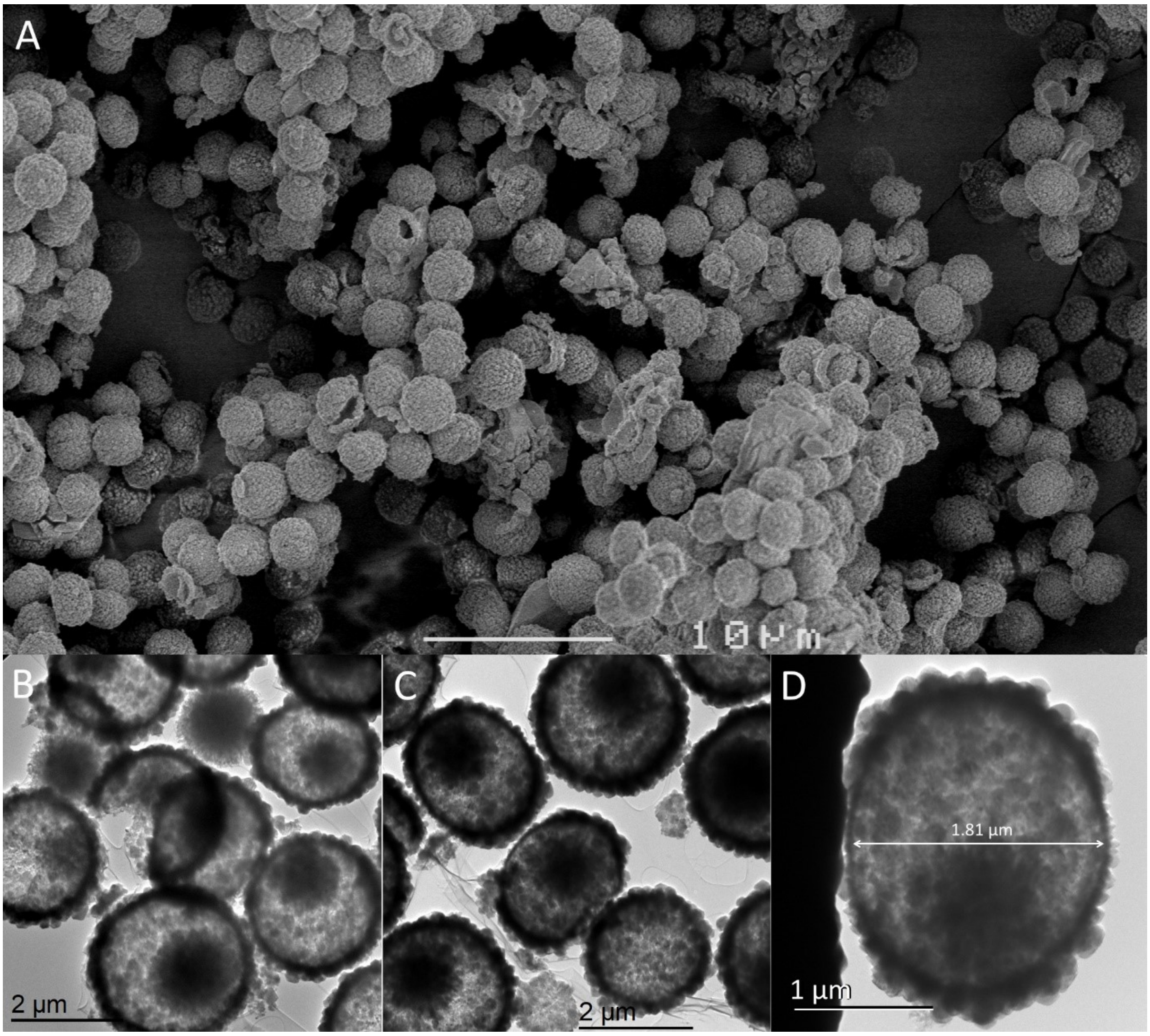

Figure S6: SEM and TEM images of the vaterite *8mM-1gL-8h* microspheroids. (A) an SEM image, and (B-D) TEM images.

pH measurements and optical images show that PSS slows down the precipitation of $CaCO_3$.

To demonstrate that PSS partially inhibits the precipitation of $CaCO_3$, the pH of 3 solutions was monitored. The first solution simply contains water (10mL) (white circle curve in Figure S7A). The second solution is an aqueous $CaCl_2$ solution (8mM, 10mL) without PSS (black square curve in Figure S7A). The third solution is an aqueous $CaCl_2$ solution (8mM, 10mL) with 1g/L PSS (blue hexagon curve in Figure S7A). At t = 0, an aqueous Na_2CO_3 solution (0.5M, 0.165mL) is injected rapidly. Note that for each of the 3 solutions, before the injection of the Na_2CO_3 solution, the pH decreases progressively due to the dissolution of CO_2(g) coming from the air. We choose to inject the Na_2CO_3 solution when the pH was 6.7. It can be observed that the injection of the carbonate solution creates a sudden increase in the pH due to the addition of CO_3^{2-} ions. In the absence of calcium ions, the precipitation of $CaCO_3$ does not take place and the pH remains stable at about 10.8 (white circle curve in Figure S6A). For the other 2 curves, the decrease in pH over time is related to the precipitation of $CaCO_3$. The precipitation is directly correlated to the acidification. It can thus be seen that qualitatively the presence of PSS slows down the acidification. This means that PSS delays the precipitation of $CaCO_3$. However, it is not possible to deduce from the pH values the amount of solid $CaCO_3$ formed because the CO_2(aq) concentration is unknown and changes over time. Figure S7(B) is complementary to the pH measurements. It shows optical images of the solution/suspension just after injection of the Na_2CO_3 solution (0.5M, 0.165mL) into the 10mL aqueous solution of $CaCl_2$ (8mM) and PSS. In the absence of PSS, the solution becomes cloudy upon injection of the carbonate. This whitish appearance is related to the precipitation of solid $CaCO_3$. Conversely, when the initial concentration of PSS is high, i.e., when the S/Ca ratio is high, the solution takes longer to cloud. It can be deduced from this that PSS delays the precipitation of $CaCO_3$. These results confirm the ones deduced from pH measurements (see Figure S7A).

A

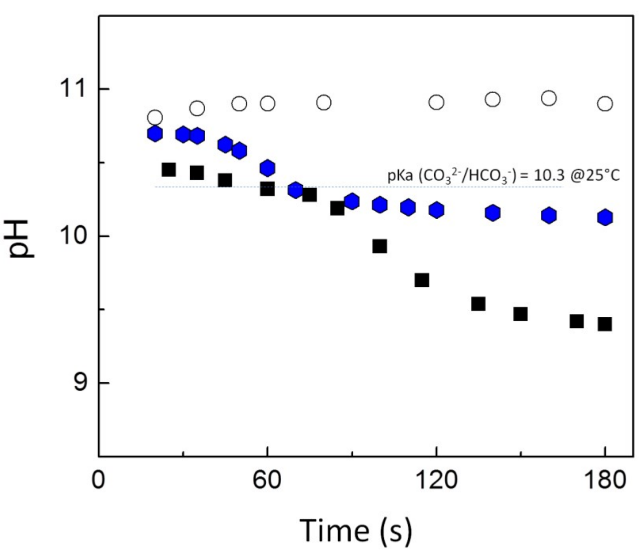

B

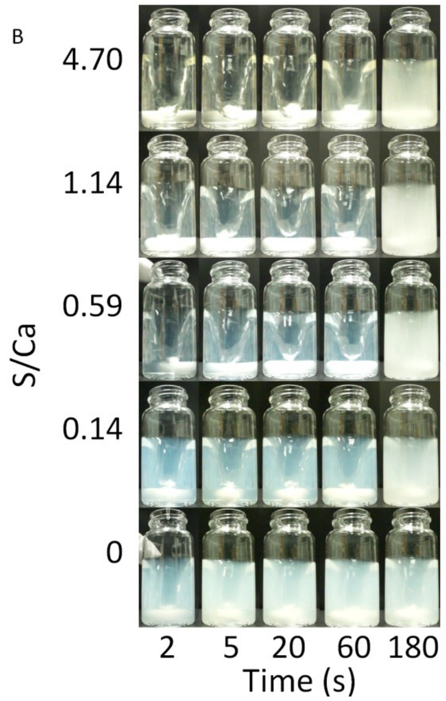

Figure S7: pH measurements and optical images show that PSS slows down the precipitation of $CaCO_3$. (A) Evolution of the pH of 3 aqueous solutions of 10mL just after the injection of a solution of Na_2CO_3 (0.5M, 0165mL) at t = 0. Before the injection, the first solution simply contains water (10mL) (white circle curve). The second solution is an aqueous $CaCl_2$ solution (8mM, 10mL) without PSS (black square curve). The third solution is an aqueous $CaCl_2$ solution (8mM, 10mL) with 1g/L PSS (blue hexagon curve). (B) Optical image of the 8mM solution just after injection at t = 0s of the Na_2CO_3 solution (0.5M, 0.165mL).

SEM images of *8mM-0.27gL-8h*.

Figure S8 shows SEM images of the *8mM-0.27gL-8h* sample. Figure S8A shows that the microspheroids have dimensions close to each other. Figure S8B shows microspheroids with a broken shell. This image emphasizes the fragility of such structures. In addition, the core of some particles is still present after 8 hours of reaction. Figures S8C and S8D are zooms of one of the microspheroids shown in image S8B. It is interesting to note that the size of the nanodomains present on the inner side of the shell is of the order of 30-50 nm, in agreement with CXDI observations (see Figure 5C(ii))

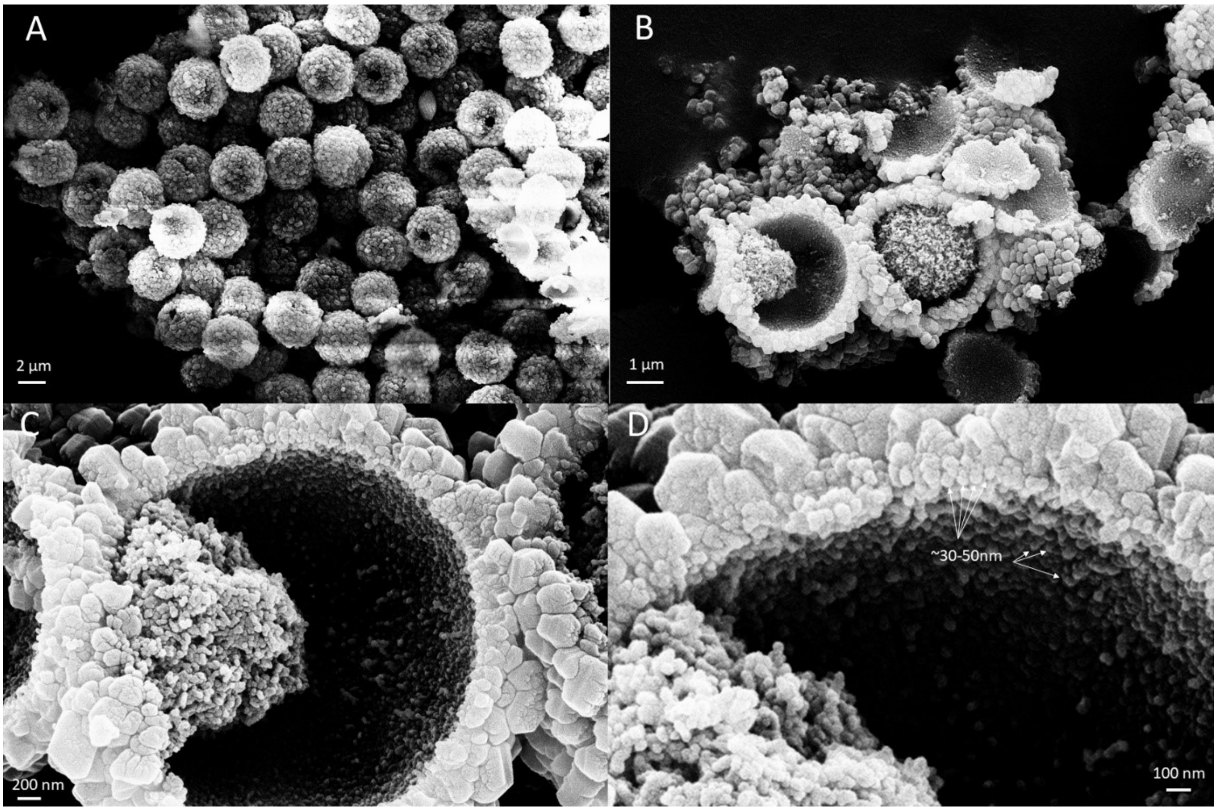

Figure S8: SEM images of *8mM-0.27gL-8h*. In D, the white arrows point nanodomains of 30-50nm.

Infrared spectroscopy shows a correlation between the growth of $CaCO_3$ and the release of PSS macromolecules.

Vaterite has four internal vibration modes located at 745 cm^{-1} (ν_4 mode), at 875 cm^{-1} (ν_2 mode), at 1076 cm^{-1} and 1088 cm^{-1} (ν_1 mode) and between 1350 and 1500 cm^{-1} (ν_3 mode).[4] The two most intense peaks of ν_3 are located at 1407 and 1430 cm^{-1}. On the Raman spectra, the ν_1 mode is the most intense and is generally used to distinguish the vaterite phase from the other phases. [5] In the infrared spectra, the ν_4 vibration mode can be used to identify the $CaCO_3$ phase. It is located at 745 cm^{-1} for vaterite, at 713 cm^{-1} for calcite, and as a doublet at 700 and 713 cm^{-1} for aragonite at 700 and 713 cm^{-1}.[6] Here, the absence of peaks at 700 cm^{-1} and 713 cm^{-1} shows that the only crystalline variety of $CaCO_3$ formed is vaterite. The presence of vibration modes at 1010 cm^{-1}, 1040 cm^{-1}, 1129 cm^{-1}, and 1177 cm^{-1} demonstrates also the presence of PSS within the vaterite. This confirms the local measurements obtained by 3D nanofluorescence (see Figure 2). It should also be noted that no $\nu_{O\text{-}H}$ vibration mode was not detected between 2700 and 3800 cm^{-1} (results not shown). This highlights the absence of hydrated amorphous calcium carbonate (ACC).[7] In figure S9A, the infrared spectra of *8mM-1gL-30min* and *8mM-1gL-24h* samples are shown. For these samples for which the average size of the vaterite crystals increases with reaction time as clearly observed by SEM, TEM, and CXDI, we see that the intensity of the PSS modes decreases between 30min and 24h of reaction. This can be understood as a release of the PSS macromolecules during the growth of the vaterite nanocrystals. In figure S9B, the infrared spectra of *30mM-8gL-1min* and *30mM-8gL-72h*. For these samples for which the growth of the vaterite crystals is inhibited by a high amount of PSS, we see that the intensity of the PSS modes does not decrease significantly between 30min and 72h of reaction. We deduce that the slower the ripening of the vaterite crystal, the slower the release of PSS from the vaterite nanocrystals to the solution. The figure S9(C) shows the infrared spectra of the samples *30mM-1gL-1min, 30mM-1gL-30min, 30mM-1gL-2h, 30mM-1gL-8h, 30mM-1gL-24h, 30mM-1gL-72h, 30mM-1gL-9days, 30mM-1gL-27days*. The absence of a peak at 713cm^{-1} confirms that the powders contain neither calcite nor aragonite. The only $CaCO_3$ polymorph is vaterite even after 27 days in solution. We conclude that the phase transformation of vaterite into calcite or aragonite is thus inhibited for a long time due to the presence of PSS whose vibration modes are located at 1010 cm^{-1}, 1040 cm^{-1}, 1129 cm^{-1}, and 1177 cm^{-1}. We observe that the intensity of these peaks decreases with reaction time. This agrees with our previous observation. When the initial PSS concentration is not too high, Ostwald ripening takes place. This leads to an increase in the average size of the vaterite crystals. This growth is correlated with a decrease in the PSS content.

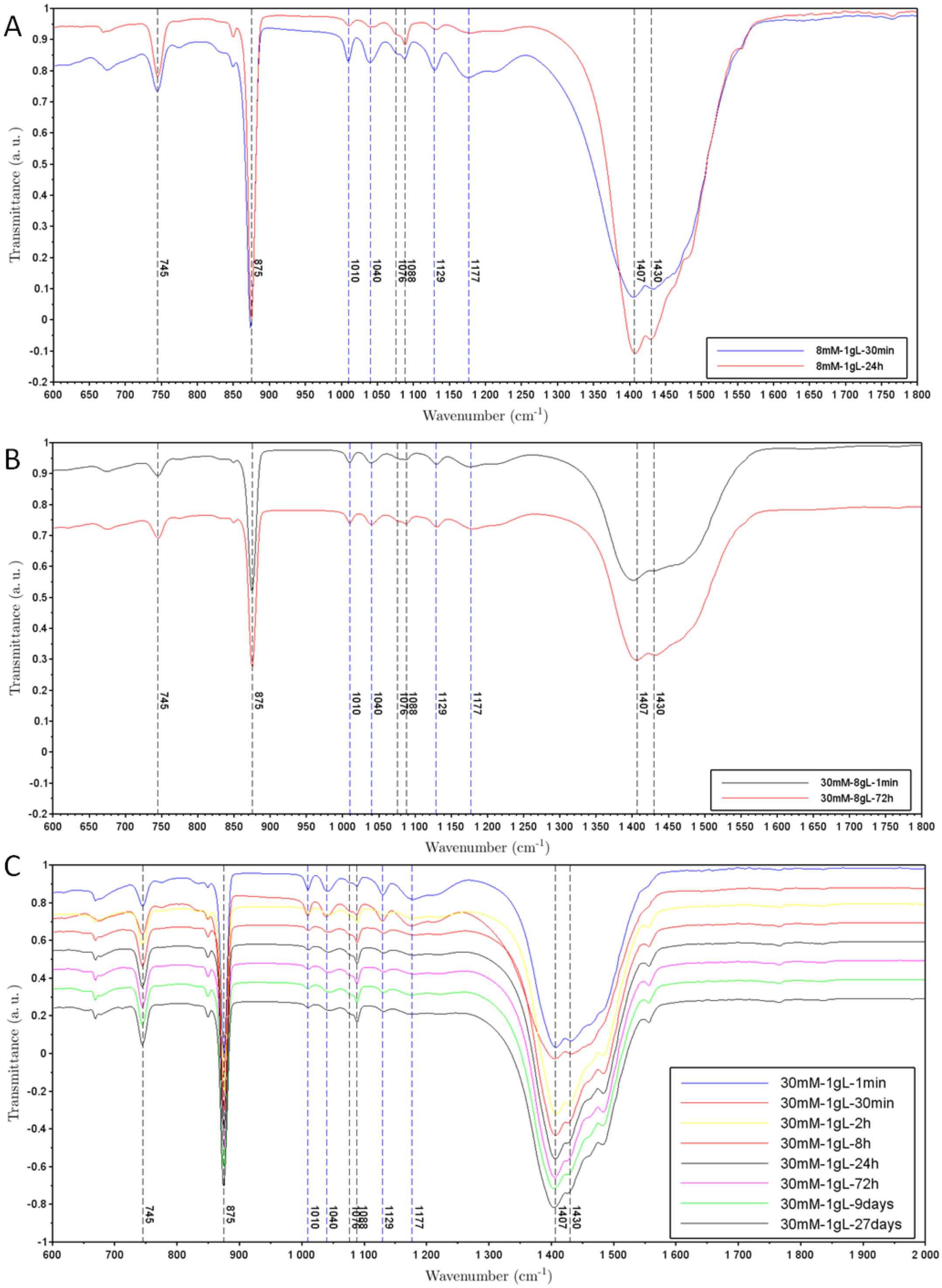

Figure S9: Infrared spectroscopy shows a correlation between the growth of $CaCO_3$ and the release of PSS macromolecules. Infrared spectra in the range 700-1800 cm^{-1} of (A) *8mM-1gL-30min* and *8mM-1gL-24h* and of (B) *30mM-8gL-1min* and *30mM-8gL-72h*. The vibration modes of vaterite are located at 745 cm^{-1}, 875 cm^{-1}, 1076 cm^{-1}, 1088 cm^{-1} and the broad band between 1350 cm^{-1} and 1550 cm^{-1} . The vibration modes of PSS are at 1010 cm^{-1}, 1040 cm^{-1}, 1129 cm^{-1} and 1177 cm^{-1}. On (B), the spectra were y-shifted to avoid their superposition and to clarify the observation. (C) Infrared spectra in the range 600-2000 cm^{-1} of the samples *30mM-1gL* obtained after reaction times from 1min to 27days.

CXDI of *30mM-1gL*

Figure S10 shows CXDI images obtained with a voxel size of 17.7nm on microspheroids of *30mM-1g/L*. After short reaction times (A and B), a shell is already formed. This means that there is already a gap between the shell and the core. The fact that the nanoparticles of the core dissolve may come from either a smaller size or a lower PSS coverage compared to the one of the shell. Besides, we believe that the nanoparticles of the shell grow because they are in contact with the solution. Because the solution is always over-saturated, i.e., never in equilibrium with the solid, Ca^{2+} and CO_3^{2-} ions from the solution can participate in the growth of the outermost first layers of the microspheres. At the opposite, the nanoparticles of the core are isolated from the solution. Their sizes are determined by the initial saturation conditions at the very early stage, i.e. when the microspheres grow. Figure B(iii) and (iv) shows that the shell contains, after 30 min of reaction, nanoplatelets with a hexagonal base. After 8h of reaction (Figure C), these nanocrystals are bigger with a bipyramidal shape ((iii) and (iv)). They tend to be oriented along the meridians. While the nanoparticles and the nanopores of the core are very small (<30nm) after 1 min and 30 min, they are far bigger after 8h of reaction. This shows that the Ostwald ripening of the core leads to the dissolution of the smallest nanoparticles and to the growth of the biggest nanoparticles. This is totally different from the *8mM-1g/L* microspheroids for which the growth does not take place in the core. In the *8mM-1g/L* synthesis, the nanoparticles of the core only dissolve with time. We explain these differences between the *8mM-1g/L* and the *30mM-1g/L* synthesis by considering that the size polydispersity of the nanoparticles of the core in the early stage is larger for the *30mM-1g/L* compared to the one of *8mM-1g/L*. Then, the initial polydispersity size increases with reaction time as shown in figures D (after 24h) and E (after 72h).

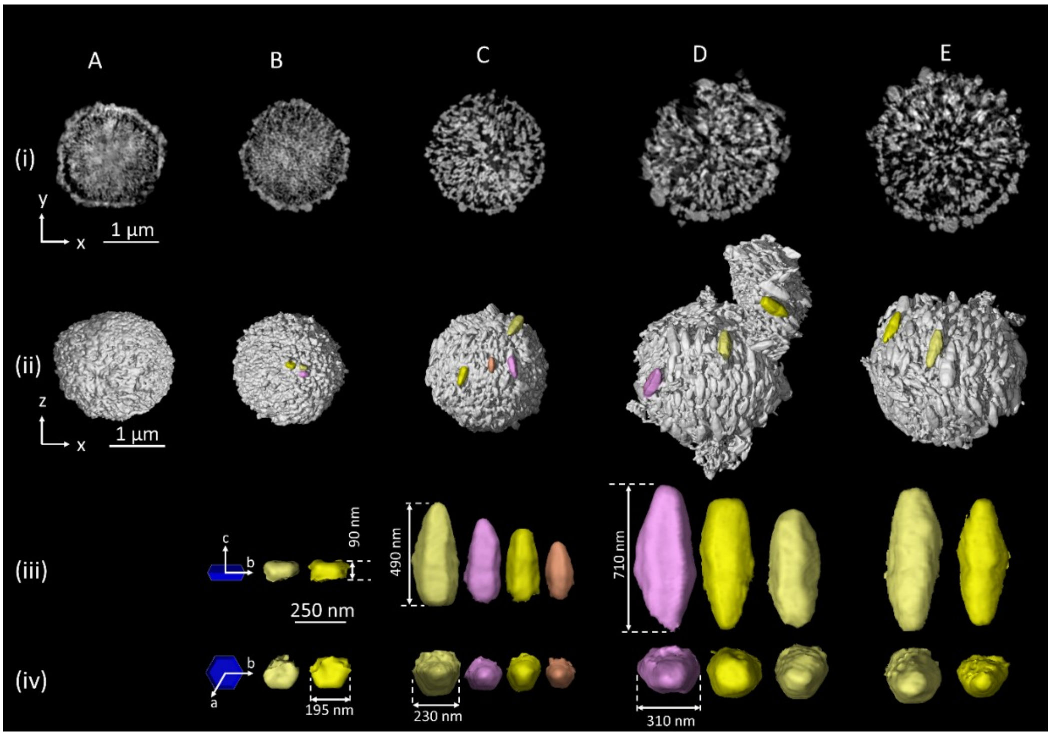

Figure S10: CXDI of 30mM-1gL. CXDI (Voxel size = 17.7 nm) on vaterite microspheres obtained from the synthesis *30mM-1g/L* after aging times of (A) 1min, (B) 30min, (C) 8h, (D) 24h, and (E) 72h. Line (i): cuts in the xy-plane passing through the center of each of the microparticles. Line (ii): 3D views observed in the xz-plane. Line (iii-iv): Vaterite crystals extracted from the microspheres and observed along (iii) [100]* and (iv) [001]*. The scale bar is 1µm for (i) and (ii), and 250nm for (iii) and (iv). In (B, iii, and iv), the blue crystallite is a theoretical bifrustum (truncated bipyramid with a hexagonal base) allowing a better understanding of crystal geometry in the first moments of aging.

The microspheroid *30mM-0.24gL-24h* is flattened at the poles

Figure S11 shows (A) an SEM image and (B-G) CXDI images of a microspheroid of *30mM-0.24gL-24h*. Figures A and B allow comparison between SEM and CXDI with the same orientation of the microspheroid. While the spheroidal shape is not visible on the SEM image (Figure S11A), it can be detected on CXDI (Figure S11B) by calculating the radius of the microspheroid, i.e. the distance of the outer surface from the center of the microspheroid. The blue region refers to radii of around 2.8µm, denoted “Pole n°1” in Figure S11B. This value is slightly lower than the average radius of the microspheroid. By looking at the opposite side, a second blue region appears, denoted “Pole n°2” in Figure S11C. Thus, the spheroidal shape is visible by CXDI. By rotating the microspheroid, the alignment of the vaterite nanocrystals along the meridians can be highlighted (Figures S11 D-F). The spheroidal 2D view shown in Figure S11G confirms this alignment even if some dispersion exists. Thus, the alignment of the nanocrystals along the meridians is linked to the spheroidal shape. This correlation can be understood by assuming that each nanocrystal has an electric dipole moment. The

minimization of the dipolar energy forces the nanocrystals to be co-aligned. In addition, as the poles can be considered as frustrated regions where the co-alignment is not possible (except if the nanocrystals are out-of-plane, which is not the case here), the growth of the nanocrystals at the poles are inhibited which tends to decrease the pole radius of the microspheroid. This is confirmed by looking inside the microspheroid (Figures S11H-I). The porosity at the poles is far more important. We can conclude that the poles are regions where the nanocrystals are dissolving.

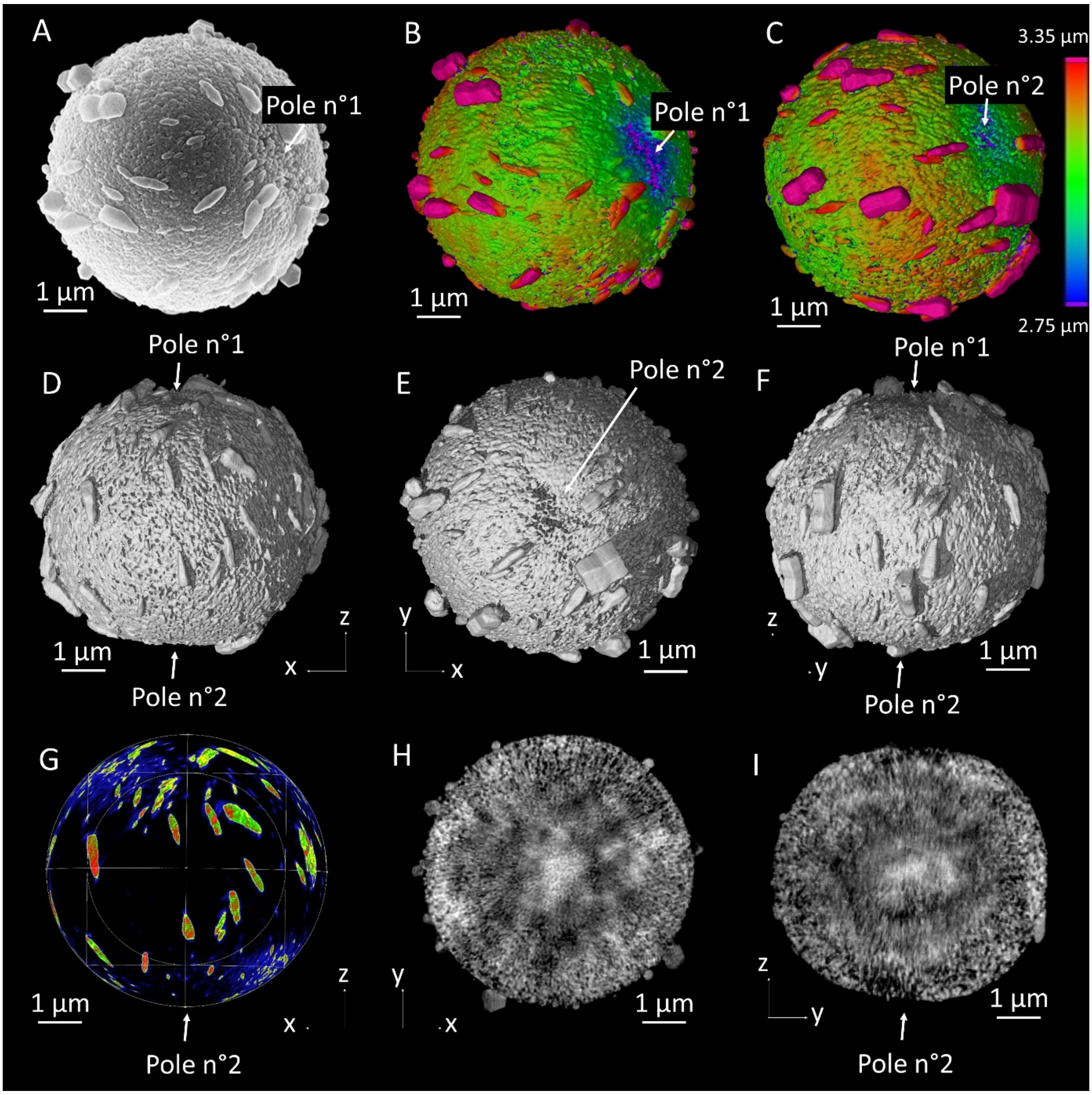

Figure S11: (A) SEM image and (B-G) CXDI images of *30mM-0.24gL-24h*. In B, the orientation of the microspheroid is similar to the one shown in A. In B and C, the colorbar refers to the radius of the microspheroid, i.e. to the distance of the outer surface of the microspheroid to the center. (G) Spheroidal 2D view. (H, I) Planar 2D view (H) in the equatorial plane and (I) in a polar plane.

2D thick slice allows better observation of the bilayered structure of the shell

The 2D views of the Figure S12 are obtained by superimposing a stack of images oriented according to z and over a thickness of (A) 12nm (1 voxel), (B) 200nm (16 voxels), and (C) 400 nm (32 voxels). In each pixel of the resulting image, the grayscale is equal to the maximum grayscale of the pixels in the image stack. The stacking of the images makes it possible to better observe the bilayered structure of the shell with on the outer part the presence of large vaterite crystals and on the inner part the presence of small crystals whose size is on the order of 30 to 50 nm.

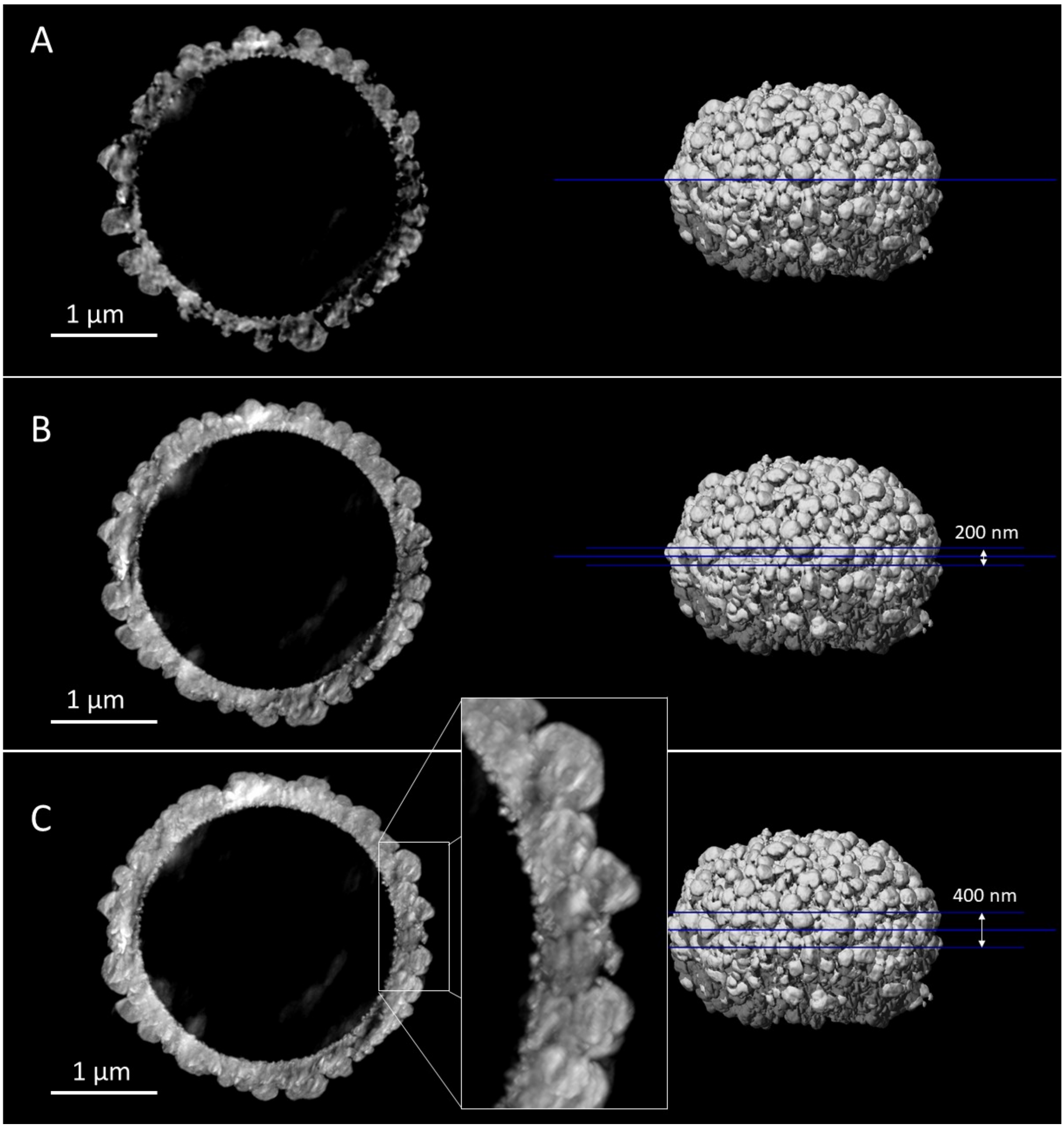

Figure S12: Stacks along the z-axis of (A) 1 image, (B) 16 images and (C) 32 images of *8mM-0.27g/L*-24h (Vx = 12.6 nm). The z-axis corresponds to the axis going through the poles.

Electron diffraction confirms the c-axis alignment of the nanocrystals

The figure S13 A shows a TEM image of a *8mM-1gL-72h* microspheroid viewed along the *xy*-plane. A TEM image of an isolated nanoparticle is shown in B and its selected area electron diffraction pattern in C. The analysis of the diffraction peak reveals that the zone axis is [12-1] (Kamhi's model). The orientation of the microspheroid in the *xy*-plane is not conducive to demonstrating that the hexagonal based bipyramidal nanocrystals are elongated along their c-axis. At the contrary, the figures S13 D shows a TEM image of an isolated nanocrystal from a *120mM-1gL-8h* microsphere elongated along the carbon membrane. In this case, electron diffraction of a selected area shows clearly that the nanocrystal is elongated along the c-axis (Figure S13 E).

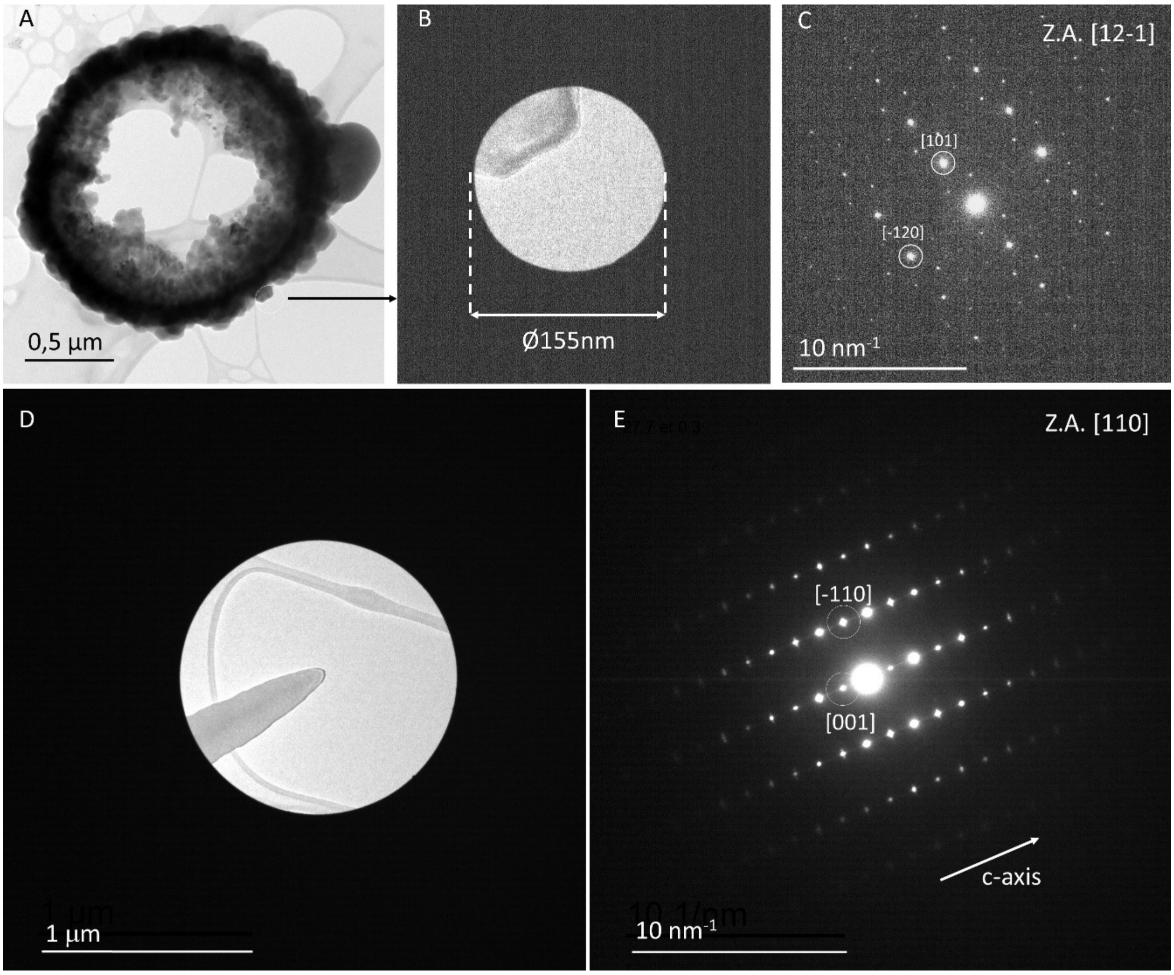

Figure S13 : (A) TEM image of a *8mM-1gL-72h* microspheroid viewed along the *xy*-plane. (B) TEM image of an isolated nanocrystal obtained by using a diaphragm of 155nm diameter and (C) its electron diffraction pattern. The zone axis is [12-1] (Kamhi's model with a = 0.413 nm and c = 0.849nm). Surstructure peaks are clearly visible. (D) TEM image of an isolated nanocrystal from a *120mM-1gL-8h* microsphere. (E) Electron diffraction pattern of the area shown in D. The zone axis is [110] confirming that the elongation of nanocrystals is along the c-axis (Kamhi's model with a = 0.413 nm and c = 0.849nm).

1. Farhadi Khouzani, M.*, et al.* Disordered amorphous calcium carbonate from direct precipitation. *CrystEngComm* **17**, 4842-4849 (2015).
2. Shen, Q.*, et al.* Properties of Amorphous Calcium Carbonate and the Template Action of Vaterite Spheres. *The Journal of Physical Chemistry B* **110**, 2994-3000 (2006).
3. Kamhi, S. On the structure of vaterite CaCO3. *Acta Crystallographica* **16**, 770-772 (1963).
4. Xu, B. & Poduska, K.M. Linking crystal structure with temperature-sensitive vibrational modes in calcium carbonate minerals. *Physical Chemistry Chemical Physics* **16**, 17634-17639 (2014).
5. Beuvier, T.*, et al.* Phase transformations in CaCO3/iron oxide composite induced by thermal treatment and laser irradiation. *J Raman Spectrosc* **44**, 489-495 (2013).
6. Vagenas, N.V., Gatsouli, A. & Kontoyannis, C.G. Quantitative analysis of synthetic calcium carbonate polymorphs using FT-IR spectroscopy. *Talanta* **59**, 831-836 (2003).
7. Rodriguez-Blanco, J.D., Shaw, S. & Benning, L.G. The kinetics and mechanisms of amorphous calcium carbonate (ACC) crystallization to calcite, via vaterite. *Nanoscale* **3**, 265-271 (2011).
